# Supplementary material for: Post-abortion care with misoprostol – equally effective, safe and accepted when administered by midwives compared to physicians: a randomised controlled equivalence trial in a low-resource setting in Kenya
Source: BMJ Open. 2017 Oct 10;7(10):e016157. doi: 10.1136/bmjopen-2017-016157 (PMC5652492; doi:10.1136/bmjopen-2017-016157)
Supplement: Supplementary data [file bmjopen-2017-016157supp001.pdf]

Appendix 1. Side-effects following treatment of incomplete abortion, self-reported by participating women.

Self-reported side effects*	Midwife	Physician	Total
**N**	409	401	810
None	201 (49%)	206 (51%)	407 (50%)
Abdominal pain	166 (41%)	139 (35%)	305 (38%)
Chills	51 (12%)	46 (12%)	97 (12%)
Nausea	32 (8%)	32 (8%)	64 (8%
Diarrhoea	27 (7%)	22 (6%)	49 (6%)
Vomiting	12 (3%)	17 (4%)	29 (4%)
Foil smelling vaginal or cervical discharge	9 (2%)	10 (2%)	19 (2%)

Data are n (%). *Several options was allowed to obtain.
