# Supplementary material for: Post-abortion care with misoprostol – equally effective, safe and accepted when administered by midwives compared to physicians: a randomised controlled equivalence trial in a low-resource setting in Kenya
Source: BMJ Open. 2017 Oct 10;7(10):e016157. doi: 10.1136/bmjopen-2017-016157 (PMC5652492; doi:10.1136/bmjopen-2017-016157)
Supplement: Author's manuscript [file bmjopen-2017-016157.draft_revisions.pdf]

BMJ Open is committed to open peer review. As part of this commitment we make the peer review history of every article we publish publicly available.

When an article is published we post the peer reviewers' comments and the authors' responses online. We also post the versions of the paper that were used during peer review. These are the versions that the peer review comments apply to.

The versions of the paper that follow are the versions that were submitted during the peer review process. They are not the versions of record or the final published versions. They should not be cited or distributed as the published version of this manuscript.

BMJ Open is an open access journal and the full, final, typeset and author-corrected version of record of the manuscript is available on our site with no access controls, subscription charges or pay-per-view fees (http://bmjopen.bmj.com).

If you have any questions on BMJ Open's open peer review process please email editorial.bmjopen@bmj.com

Post-abortion care with misoprostol - equally effective, safe and accepted when administered by midwives compared to physicians: a randomised controlled trial in a low-resource setting in Kenya.

Journal:	*BMJ Open*
Manuscript ID	bmjopen-2017-016157
Article Type:	Research
Date Submitted by the Author:	30-Jan-2017
Complete List of Authors:	Makenzius, Marlene; Karolinska Institutet Department of Public Health Sciences, Global Health (IHCAR)<br>Oguttu, Monica; Kenya Medical Education Trust - KMET<br>Gemzell, Kristina; Karolinska Universitetssjukhuset, Dept. of Women´s and Children´s Health<br>klingberg-allvin, marie; Hogskolan Dalarna Akademin Utbildning Halsa och Samhalle<br>Odero, Theresa; College of Health Sciences, School of Nursing Sciences, University of Nairobi<br>Faxelid, Elisabeth; Karolinska Institutet Department of Public Health Sciences
**Primary Subject Heading**:	Global health
Secondary Subject Heading:	Medical education and training, Obstetrics and gynaecology, Sexual health, Public health, Reproductive medicine
Keywords:	EDUCATION & TRAINING (see Medical Education & Training), Community gynaecology < GYNAECOLOGY, Reproductive medicine < GYNAECOLOGY, Health policy < HEALTH SERVICES ADMINISTRATION & MANAGEMENT, Maternal medicine < OBSTETRICS

SCHOLARONE™ Manuscripts

TITLE

Post-abortion care with misoprostol - equally effective, safe and accepted when administered by midwives compared to physicians: a randomised controlled trial in a low-resource setting in Kenya.

Short title: Post-abortion care with misoprostol: A randomised controlled trial in a low-resource setting, Kenya

AUTHORS

***Marlene Makenzius (MM),** Postdoctor monitoring the RCT at site, PhD, MPH, CNM
Dept. of Public Health Sciences Global Health (IHCAR), Karolinska Institutet
Postal address: Tomtebodavägen 18A, SE-171 77 Stockholm, Sweden
Phone: +46 761131 076, +254 7342 47498
marlene.makenzius@folkhalsomyndigheten.se; marlene.makenzius@ki.se

Monica Oguttu (MO), PI, Director at KMET, PhD, RM, RN
Kenya Medical Education Trust - KMET
Postal address: Tom Mboya Estate, P.O. Box 6805 – 40103, Kisumu, Kenya
+254 701 839 115 or 721 845948

Kristina Gemzell-Danielsson (KMD), Professor, MD, PhD, Chair Div. of Obstetrics and Gynecology
Dept. of Women´s and Children´s Health, Karolinska Institutet
Postal address: WHO-centre, C1:05, Karolinska University Hospital, SE-171 76 Stockholm, Sweden
Phone: +46 8 517- 72128 (or -79539)

Marie Klingberg-Allvin (MKA), Professor/Pro Vice-Chancellor, PhD, RM, RN
School of Education, Health and Social studies, Dalarna University
Postal address: Dalarna University, SE-791 88 Falun, Sweden

Theresa M A Odero (TO), PhD-stud, MSc, RM, RN
College of Health Sciences, School of Nursing Sciences, University of Nairobi
Postal address: P O Box 19676, code 00202 KNH, Nairobi, Kenya

Elisabeth Faxelid (EF), PI, Sweden), Prof., PhD, RM, RN
Dept. of Public Health Sciences Global Health (IHCAR), Karolinska Institutet
Postal address: Tomtebodavägen 18A, SE-171 77 Stockholm, Sweden

*Corresponding author

I Marlene Makenzius The Corresponding Author of this article contained within the original manuscript which includes any diagrams & photographs within and any related or stand alone film submitted (the Contribution") has the right to grant on behalf of all authors and does grant on behalf of all authors, a licence to the BMJ Publishing Group Ltd and its licencees, to permit this Contribution (if accepted) to be published in the BMJ and any other BMJ Group products and to exploit all subsidiary rights, as set out in our licence set out at: http://www.bmj.com/about-bmj/resources-authors/forms-policies-and-checklists/copyright-open-access-and-permission-reuse. Please tick one or more boxes as appropriate:

YES, I am one author signing on behalf of all co-owners of the Contribution.

For peer review only - http://bmjopen.bmj.com/site/about/guidelines.xhtml

Author's contribution

The lead author for this manuscript was Marlene Makenzius

Study design: Kristina Gemzell Danielsson, Elisabeth Faxelid, Marie Klingberg Allvin, Monica Oguttu and Theresa Odero
Literature search: Marlene Makenzius
Data Collection: Marlene Makenzius, Monica Oguttu and Theresa Odero
Data analysis: Marlene Makenzius, cross-checked by a senior statistician, Ulla Romild, at the National Agency of Public Health in Sweden.
Data interpretation: Marlene Makenzius Kristina Gemzell Danielsson, Elisabeth Faxelid, Marie Klingberg Allvin, Monica Oguttu and Theresa Odero.
Writing: Marlene Makenzius
Editing and proof-reading: Kristina Gemzell Danielsson, Elisabeth Faxelid, Marie Klingberg Allvin, Monica Oguttu, Theresa Odero, and Scribendix (language editing).

ABSTRACT

Objective To assess the effectiveness of midwives administering misoprostol to women with incomplete abortion, compared with physicians. Secondly to assess contraceptive uptake in post-abortion care (PAC).

Design A multicentre randomised controlled equivalence trial. The study was not masked.

Settings Gynaecological departments in two hospitals in a low-resource setting, Kenya.

Population Women (n=1094) with incomplete abortion in the first trimester, seeking PAC during 1 June 2013 to 30 August. Participants were randomly assigned to receive treatment from midwives or physicians. 409 and 401 women in the midwife and physician groups, respectively, were included in the per-protocol analysis.

Interventions 600µg misoprostol orally, and contraceptive counselling by a physician or midwife.

Main outcome measures Complete abortion not needing surgical intervention within 7–10 days. Secondary, women's acceptability of the method, and contraceptive uptake. The main outcome was analysed on the per-protocol population with a generalised estimating equation model. The predefined equivalence range was –4 % to 4%. Secondary outcomes were analysed descriptively.

Results The proportion of complete abortion was 94.8% (768/810), 390 (95.4%) in the midwife-group, and 378 (94.3%) in the physician-group. The proportion of Incomplete abortion was 5.2% (42/810), similarly distributed between midwives and physicians. The model-based risk difference for midwives versus physicians was 1.0% (–4.1 to 2.2). Most women felt safe (97%; 779/799), 93% (748/801) perceived the treatment as expected/easier than expected. After contraceptive counselling the uptake of a contraceptive method occurred in 76% (613/810). No serious adverse events were recorded.

Conclusions Treatment of incomplete abortion with misoprostol when provided by midwives is as effective, safe, and accepted as when administered by physicians in a low-resource setting. Systematically provided contraceptive counselling in abortion-care is effective to mitigate unmet need for contraception.

The trial was registered at ClinicalTrials.gov, NCT01865136

For peer review only

Strengths

- Systematically trained midwives in a low-resource setting can independently diagnose incomplete abortion, and successfully treat women with misoprostol.
- The misoprostol-treatment is highly accepted among the treated women.
- Uptake of a contraceptive method is high when contraceptive counselling is systematically provided composed with a wide range of contraceptive choices in PAC. Mitigating the unmet need for contraception could be the key to reducing numbers of subsequent unintended pregnancies and unsafe abortions, as well as maternal morbidity and mortality.
- In areas with shortages of physicians and scarce resources, midwives would greatly increase women's access to safe and highly accepted PAC.

Limitations

- Characteristics of study participants were representative of women in fertile ages in Kisumu County, they might not be representative of women living in the most remote rural areas of this county.
- District hospitals are usually more advanced regarding staffing and resources and might therefore not be representative of the small health dispensaries located in remote areas of the region.
- Lacking long-term follow-up on contraceptive adherence after PAC.

INTRODUCTION

Globally, every fourth pregnancy in 2010–2014 ended in induced abortion, and corresponds to an average of 56 million induced abortions annually.[1] Since 1990, the annual abortion rate has declined in high-income countries, yet remains the same in low-income countries.[1] Consequently, the annual abortion rate is now higher in low-income countries (37 per 1,000 women) than in high-income countries (27 per 1,000 women).[1] In Kenya, the rate of induced abortion in 2012 was estimated to occur in 48 in 1000 women of reproductive age (15–49 years), resulting in 30 abortions per 100 births.[2]

Although induced abortion is medically safe when done in accordance with recommended guidelines[3], many women undergo unsafe procedures that put their health and life at risk.[2,4,5] Unsafe abortion is defined by WHO as a procedure performed under conditions that fail to meet the minimal medical standards and/or by untrained persons.[6] The majority of unsafe abortions occur in low-income countries where induced abortion is restricted, and is a leading cause of death among young women in sub-Saharan Africa.[1,2,4,5] Under the 2010 Kenyan constitution, abortions may be induced if the woman needs emergency treatment or her life or health is in danger.[7]

It is estimated that seven million women in low-income countries are treated for complications from unsafe abortions every year and at least 22,000 die from abortion-related complications every year.[1] The costs for post-abortion care (PAC) constitute a significant financial burden on public healthcare systems in these countries.[4,8-10] In Kenya, an estimate of 120,000 women are treated for

For peer review only - http://bmjopen.bmj.com/site/about/guidelines.xhtml

For peer review only

complications resulting from unsafe abortion annually[2], and 35% of maternal deaths are attributed to unsafe abortions.[2] The proportion of severe complications such as high fever, sepsis, shock, and organ failure is high (37%), and is most common among women who have interfered with the continuation of the pregnancy (58%), divorced women (56%), and women aged 10–19 (45%).[2]

In Kenya, 71% of women seeking PAC did not use a contraceptive method prior to becoming pregnant.[11] The high unmet need for contraception helps explain the prevalence of induced abortions in Kenya.[2,11-13]

PAC is an integral component of comprehensive abortion care (CAC), and is often used in settings where abortion is legally restricted.[14] Essential elements are community and service provider partnerships, counselling, treatment of incomplete and unsafe abortion, contraceptive services, and linkages to reproductive and other health services.[14] The prostaglandin E_1 analogue misoprostol is an effective drug to treat incomplete abortion in the first trimester, in PAC[15], and no significant differences were found in the effectiveness between misoprostol and manual vacuum aspiration (MVA) for the treatment of incomplete abortion.[16,17] Misoprostol simplifies the procedure, compared to MVA which is more intrusive.[15]

The use of MVA and medical abortion is growing in Kenya; however, other less safe procedures remain common.[18] There is also a mismatch between where maternal mortality exists and where health workers are, suggesting critical inequities in Kenya in the availability for high-quality PAC.[18] Shifting/sharing tasks may increase access to and availability of services without compromising performance or patient outcomes and may be cost-effective.[19-23] Trained nurses have been proven to provide safe medical abortion in Nepal[24], and trained midwives to treat incomplete abortion in Uganda[25] by using misoprostol. However, regulations restrict misoprostol prescription and supervision to physicians, and the high proportion of complications and high fatality rate indicate that unsafe abortions continue to pose a major public health challenge in low-income settings.[1,2]

There is an urgent need to provide timely and effective treatment at health facilities.[1-5,18,21,26,27] PAC service providers must also ensure that women receive contraceptive counselling and effective contraceptives to prevent a subsequent unintended pregnancy.[1,11-13,18,21] However, ensuring the availability of medical equipment, personnel, knowledge and skills, is a tremendous challenge in resource-limited economies.[20,21,27] This restricts women's access to proper care, and forces them to obtain unsafe abortions.[18,21,28] Renner et al.[22] (2013) found limited evidence indicates that trained mid-level providers may effectively and safely provide first trimester termination of pregnancy, including incomplete abortion services. Data are limited by the scarcity of RCTs and biases of the cohort studies.

For peer review only - http://bmjopen.bmj.com/site/about/guidelines.xhtml
For peer review only

The overarching aim with this trial was to assess the effectiveness and safety of midwives administering misoprostol to women with incomplete abortions, compare to physicians in a low-resource setting. Second, we aimed to investigate the women's acceptance of the treatment as well as their uptake of a contraceptive method in PAC.

METHODS

The project was conducted at the Jaramogi Oginga Odinga Teaching and Referral Hospital (JOOTRH) and Kisumu County Hospital (KCH) in Kisumu County, western Kenya. Compared to Kenya, this region has the lowest mean age (16.4 years) at first sexual intercourse, highest rate of teenage pregnancy and the highest HIV prevalence.[18] Kisumu is the third largest city in Kenya and has a population of 500,000. The project was implemented in the gynaecological ward within the Department of Obstetrics and Gynaecology at the two facilities. Altogether the two facilities admit around 26 women with incomplete abortions per month. Midwives (n = 19) and physicians (n = 18) included in the project, Table 1, underwent a standardised training.[29-30]

The study was a multicentre randomised controlled trial, and the study protocol and trial followed the CONSORT guidelines for non-inferiority and equivalence randomised trials.[31]

The healthcare facilities selected for inclusion were equipped by the project to provide basic and emergency obstetric services and were estimated to have sufficient staff and resources required for the trial. The procedure and the instruments were pilot tested at the two hospitals before the study started. The first two months was set to a run-in phase, in which the facilities were given time to set the routine among staff for screening, enrolment, and follow-up, as well as to emancipate space to store a large number of protocol-records safely, and to provide counselling in a secluded room. Minor revisions were made to the instruments and the recruitment procedure.

Patient involvement

The study was designed and coordinated by researchers at the Department of Public Health Sciences, Global Health (IHCAR), and Women's and Children's Health, Karolinska Institutet, Stockholm, Sweden; Kisumu Medical Education Trust (KMET); University of Nairobi; and Moi University, Eldoret, Kenya; and at JOOTRH and KCH in Kisumu, Kenya.

Research question and outcome measures was constructed by the researcher, and health care providers at the study facilities who were experienced in PAC, in order to strengthen the validity. The preliminary results was continuously reported during the study period to the two facilities by the study coordinator to encourage the provider's work, who also used the information to encourage the

For peer review only - http://bmjopen.bmj.com/site/about/guidelines.xhtml

participating women. The outcome and acceptability of the intervention was evaluated by the provider at follow-up, and the women were advised to seek care ahead the appointment if they obtained unexpected symptoms/burden.

We obtained ethical approval from the JOOTRH Ethical Review Committee (diary number ERC42/13), and the Swedish regional Ethics Committee in Stockholm (2013/902-31/1). All the patients gave written informed consent. The participants could withdraw from participation at any time without consequences. Consent forms were translated into the local languages Kiswahili and Luo and back translated into English to ensure accurate translation. The form was read to each participant and those unable to write marked the form with a thumb print, and a witness signed to indicate that the study had been explained to the participant and that she understood and accepted to participate.

Inclusion and exclusion criteria

Inclusion criteria were women with signs of incomplete abortion (i.e., bleeding, contractions and lower abdominal pain during pregnancy, open cervical os, and partial expulsion). Exclusion criteria were complete abortion, known allergy to misoprostol, a uterine size estimated over 12 weeks of gestation, suspected ectopic pregnancy (severe abdominal pain), unstable haemodynamic status, and signs of pelvic infection or sepsis.

Randomisation and masking

Eligible women who consented to participate were randomly allocated to a midwife (intervention) or standard care with a physician (control) for diagnosis and treatment. The randomisation was done in blocks of eight and was stratified for study site. We used a computer random number generator to generate a list of codes from 1 to 890 and each code was linked to one of the two study groups. Sequentially numbered, opaque, sealed envelopes, each containing a random allocation, were prepared at the coordinating department of KMET, and later opened in consecutive order by the research assistants after obtaining written consent. Data management was organised locally at the coordinating department at KMET, Kisumu.

The study coordinator checked protocols for accuracy, corrected protocols after discussion with research assistants, and did continuous process evaluation. Study protocols were collected and data was entered continuously throughout data collection. The providers received support and guidance throughout the study period by the study coordinator. The study was not masked to the study participants or providers.

For peer review only - http://bmjopen.bmj.com/site/about/guidelines.xhtml

Procedures

Eligible providers for participation were midwives and physicians involved in PAC at the two facilities. The healthcare providers were trained in a 5-day training program according to Kenyan and the WHO standardised PAC training module.[29-30] The training was both theoretical and practical and ongoing facility-based continuous medical education occurred throughout the project period in order to cover all/new staff. This training included knowledge on diagnosing incomplete abortions, treatment with misoprostol and MVA and post-abortion contraceptive counselling as well as interview technique.[3,14,19,29-30]

Women admitted with signs of incomplete abortion were screened based on self-reported last menstrual period and symptoms. Two midwives at each facility were trained to be research assistants in the study and were responsible for screening and enrolment of participants and to oversee and monitor supply in stock (misoprostol and contraceptives such as condoms, combined pills, Depo-Provera, hormonal implants and intra uterine devices [IUD]. The research assistants were responsible for the follow-up visits including assessment of abortion status at follow-up, and provided MVA kits when necessary, all in accordance to local and international guidelines.[3,29]

Eligible women who consented to participate were randomly allocated to the midwife or physician group. The clinical assessment included anamneses and physical examination (Table 2); the last menstrual period, obstetric and gynaecological history, pelvic examination including any signs of genital infections, cervical status, bleeding, and size of the uterus. Ultrasound was not used.

Each participant was given one single dose of 600 µg misoprostol orally. Additionally, they were offered analgesics (ibuprofen or paracetamol) and oral antibiotics given according to local guidelines for PAC. For monitoring purposes, participants were advised to stay at the clinic for 4 to 12 hours after swallowing the misoprostol tablets. Before discharge, all women were offered contraceptive counselling and provided with a contraceptive method if they wanted, and given a follow-up appointment after 7–10 days.

Participants received detailed information about bleeding, pain expected, abnormal symptoms after treatment (severe pain, fever, and foul smelling vaginal discharge), and were informed about the importance of seeking care if such symptoms occurred. Adverse events were recorded. The women were offered reimbursement for travel costs as an encouragement to come for follow-up. All women received an SMS reminder two days before the scheduled follow-up appointment. Women who did not show up were contacted by the research assistant using a cell phone provided by the project, and the woman was given a new appointment and encouraged to attend.

For peer review only - http://bmjopen.bmj.com/site/about/guidelines.xhtml

Outcomes

The pre-specified primary outcome was complete abortion, requiring no further medical or surgical intervention, Table 3. The following secondary outcomes were assessed and self-reported by the patient (Table 4):

(i) A symptom diary card was used to assess daily bleeding in relation to normal menstrual bleeding,
(ii) pain following the treatment,
(iii) unscheduled visits at a healthcare facility and the reason,
(iv) women's acceptability of the treatment was recorded by three questions, and
(v) contraceptive uptake was recorded by three questions (Table 5).

Statistical analyses

The sample size was calculated with the objective of showing two-sided equivalence assuming that the rate of incomplete abortions could be 4% and would apply to both types of providers.[31] A pre-defined acceptable difference in completion rate between the two providers ranged from –4% to +4%. To establish equivalence with a power of 80% and two-sided 95% CI, the required sample size was 400 per group. Compensation for about 10% loss to follow-up gave a total sample size of 888. Background characteristics and secondary outcomes are presented with descriptive statistics, as per the pre-protocol.

Analysis of the primary outcome (complete abortion) was made by per-protocol analysis with a generalised estimating equation model to estimate the risk difference between the two providers with (two-sided) 95% CI. The intention-to-treat population was considered to be all randomised women with data for the primary outcome. If the CI of the risk difference between the two groups fell within the predetermined margin of equivalence (-4% to +4%), the two types of providers were considered equivalent. P values of 0.05 or less were considered statistically significant. Additionally, we adjusted for the following covariates: facility, age (<25 vs ≥25 years), marital status (single vs married or cohabiting), education (none, primary 1–4, or 5–8 vs secondary or tertiary), number of previous pregnancies (1 vs >1), and live births (0 vs ≥1). We estimated the adjusted risk difference as the predicted risk difference at the average of all included covariates. Equivalence between the two study groups can be stated if the 95% CI of the risk difference lies completely within the predetermined limits of equivalence (–4% to 4%). Safety parameters of the procedure were viewed descriptively without any formal statistical testing. The study protocol was not changed after the run-in period. All data were entered and cleaned in IBM SPSS Statistics for windows version 22.0, and we did all the statistical analysis within this software.

For peer review only - http://bmjopen.bmj.com/site/about/guidelines.xhtml

For peer review only

The trial is registered at ClinicalTrials.gov, number NCT01865136.

Role of the funding source

The authors designed this an investigator-initiated trial, in collaboration with healthcare providers and management at the study setting. The funders did not play any role in the study design or in the collection, analysis, and interpretation of data. All authors had full access to the data, and the first author regularly reported preliminary results to the whole research team. The corresponding author had final responsibility for the decision to submit the manuscript for publication.

RESULTS

From 1 June 2013 to 31 May 2016, we assessed 1094 women with signs of incomplete abortion for eligibility for participation in this study. Of these, 201 were ineligible, and 3 declined to participate. We randomly assigned 890 women to the midwife group (n = 446) and physician group (n = 444). A total of 31 women were excluded, of which 13 had been allocated to the midwife group (7 withdrew consent at follow-up and 6 were classified as protocol violations) and 18 had been allocated to the physician group (9 withdrew consent at follow-up, 5 were classified as protocol violations, and 3 had a gestational age of >12 weeks). A total of 49 women were lost to follow-up (24 in the midwife group and 25 in the physician group; Figure 1). Finally, a total of 810 women, 409 in the midwife group and 401 in the physician group, received misoprostol treatment with misoprostol and were included in the per-protocol analysis.

Among the providers, the midwives had longer work experience than the physicians; on the other hand, the physicians were more experienced than the midwives in PAC (Table 1).

The baseline characteristics such as sociodemographic background and reproductive history were similarly distributed between the two groups (Table 1). The mean duration of gestational age based on clinical examination was 9.6 weeks (range 1–12 weeks; Table 2).

Sub-analyses showed slightly higher proportions of young (< 25 years), single, unemployed, and nulliparous women among those who dropped out of the study compared to those who came for the follow-up visits. There were no differences in gestational age or education levels between the two groups. None of the results of the sub-analyses were statistically significant (p > 0.05).

Main outcomes

The overall proportion of complete abortion was 94.8% (768/810). As shown in Table 3, 390/409 (95.4%) women in the midwife group and 378/401 (94.3%) women in the physician group had

For peer review only - http://bmjopen.bmj.com/site/about/guidelines.xhtml

complete abortions (Table 3). The model-based risk difference for midwife versus physician group was 1.0% (–4.1 to 2.0). There were 42/810 incomplete abortions (5.2%), 19/409 (4.6%) in the midwife group and 23/401 (5.7%) in the physician group. Among the 42 women with incomplete abortions, 25 were treated with MVA and 17 were treated with repeated dosage of misoprostol after they completed the follow-up assessment. Among the 25 women who obtained MVA, 2 were treated with a repeat dosage of misoprostol prior to MVA. No serious adverse events were recorded.

Most women (690; 86%) reported bleeding less than or the same as normal menstrual bleeding after treatment, and the mean number of bleeding days was 4.1 (SD 2.0; range, 1–14). A majority (564/806; 70%) of the women reported no or mild pain after treatment, and 6% (46/806) women reported unscheduled visits. Vaginal bleeding and abdominal pain were reported as reasons for the unscheduled visits (Table 4). There were no differences in the secondary outcomes between the groups. 407/810 women reported none symptoms following the treatment, while 402/810 women reported symptoms; abdominal pain, chills, nausea, diarrhoea, vomiting, foil smelling vaginal and/or cervical discharge, and were similar in both groups (Appendix 1).

Secondary outcomes

Table 4 shows that most women perceived the treatment to be as expected or easier than expected (748/801; 93%), and 97% felt safe during the procedure (779/799). Additionally, 95% (761/799) women stated that they would recommend the treatment to a friend.

Contraceptive counselling was provided to 789 of the 810 (98%) women; of these, 613 (76%) accepted a contraceptive method of their own choice (Table 5). The following choices of contraceptive methods were offered, and distributed as follows: injectable (236/613; 39%), pills (166/613; 27%), condoms (151/613; 25%), hormonal implant (45/613; 8%), IUD (8/613; 1%), and permanent contraception (1/613; 0.16%).

DISCUSSION

Our finding shows that treating women with incomplete abortion (≤12 weeks of gestation), with misoprostol was found to be equally effective and safe when performed by midwives or physicians in a low-resource setting in Kenya. In addition, the treatment is highly accepted among the treated women (93%), and the uptake of a contraceptive method is high (76%) when contraceptive counselling is systematically provided in post-abortion care (PAC).

For peer review only - http://bmjopen.bmj.com/site/about/guidelines.xhtml

For peer review only

The socio-demographic characteristics of the women strengthen the external validity, i.e. heterogeneous, though overall low socioeconomics similar to those of the population in the county.[18,20.] The weakness of the study. First, the midwives had more years of professional experience than the physicians, but the physicians were more experienced in PAC. However, all the providers within the two facilities received the same training in the treatment of incomplete abortion. Secondly, although the socioeconomic and demographic characteristics of study participants were representative of women in fertile ages in Kisumu County, they might not be representative of women living in the most remote rural areas of this county. Third, district hospitals are usually more advanced regarding staffing and resources and might therefore not be representative of the small health dispensaries located in remote areas of the region. Fourth, both types of providers worked in the same hospital and in the same environment. We cannot rule out interactions between the two provider groups, which might have led to a convergence of outcome. Fifthly, drop-outs to follow-up was a challenge.

Though we encouraged women to come for follow-up by paying for the local transport, sent SMS reminders ahead of the scheduled appointment, and called the patient if they did not show up, 49 of the 859 women did not come for the follow-up. Some of the women did not have a phone and were thus not reachable, and some of the women who were contacted by phone did not find it necessary to attend the follow-up, as they considered themselves to have recovered without any side effects. Loss to follow-up could also be related to abortion stigma, which is present within the current county and other settings where abortion is restricted.[21] Women seeking PAC have previously reported shame and guilt, and they don't want to be associated with the abortion clinic and its activities once the abortion issue is completed.[21] Therefore, in coherence with Osur et al.[21] (2013), instead of considering drop-outs criteria as bias, this might rather be an important reflection of the stigma surrounding abortion.

The overall complete abortion rate was 94.8%, which is comparable with the results from other studies that have used the same regimen (94.5–96.7%)[16,17,24,25] In our study, ultrasonography was not available in any provider, emphasizes that the safety of using misoprostol for treatment of incomplete abortion, in low-resource settings, which can be performed by trained midwives without the routine use of ultrasonography before or after abortion.

Of the women incomplete abortion (42/810), 25 were treated with MVA and 17, with repeated dosage of misoprostol, similarly distributed between the two provider types. Repeat dosage of misoprostol is apparently an alternative for MVA when the first dosage of misoprostol has failed. This

For peer review only - http://bmjopen.bmj.com/site/about/guidelines.xhtml

could be compared with results from a similar study from Uganda where all women with incomplete abortion were treated with MVA (36/955).[25]

A notable additional finding was the frequency of women with sepsis, which was a criterion for ineligibility in this study (47/1094). This proportion is higher, that has been seen in other African countries.[2] This might be an important reflection of the magnitude of unsafe abortion and the immense poor state of reproductive health access for women in Kenya. There is no doubt that there is room for improving timely and accessible CAC services, in particular, PAC with contraceptive counselling, in Kenya.

Although these results clearly show that misoprostol is a simple, efficient and desirable method, which is highly accepted by women, implementation of PAC in Kenya is hampered by the lack of national clinical guidelines.[18,23] Laws and policies governing access to safe abortion in Africa vary, ranging from very restrictive, such as in Kenya, where abortion is permitted only to save the life of the mother, to liberal, like in South Africa, where abortion is permitted through the 12 week of pregnancy or later.[1] Stigmatizing attitudes and beliefs surrounding abortions in the society[21], not least among healthcare providers[28], contribute to women's delay in seeking timely care for abortion-related complications.

Kenya has a policy framework to prevent maternal mortality. However, this policy framework excludes unsafe abortion, which is addressed through the provision of PAC.[7,18,23] In 2012, the Ministry of Health (MH) developed clinical guidelines on prevention of morbidity and mortality from unsafe abortions in Kenya, but the document was arbitrarily withdrawn in 2013. [18,23] A task force was commissioned by the Director of Medical Services to consult and revise the guidelines, and the work was finalised in September 2015, but since then, signing of the document has stalled. Simultaneously, women seeking treatment for complications arising from unsafe abortions is on the rise.[1,18] Continued delay to sign the clinical guidelines is exposing thousands of women and girls to extensive morbidity and mortality.

New guidelines may not only contribute to increase the quality and effectiveness to treat complications of unsafe abortion, it may also encourage the prevention of unintended pregnancy.[1,18] The current result shows that systematically provided contraceptive counselling to PAC-seeking women is feasible and effective, as three out of four women accepted a contraceptive method. This indicates a pronounced need for concerted efforts to urgently reach women who have an unmet contraceptive need by giving them access to post-abortion contraceptive counselling and methods.

For peer review only - http://bmjopen.bmj.com/site/about/guidelines.xhtml

Mitigating the unmet need for contraception could be the key to reducing numbers of subsequent unintended pregnancies and unsafe abortions, as well as maternal morbidity and mortality.

Future research needs to address long-term follow-up on contraceptive adherence after PAC. There is also need to address underlying causes of morbidity and mortality from unsafe abortion because unsafe abortion is not merely a medical issue, but apathy and disdain towards women also play an important role.[26] In addition, the gap between poor and rich populations' remains, where affluent women have better access to safe abortion, regardless of whether or not abortions are legal.[1,18,21] Unsafe abortion has also significant consequences that are less widely recognized, such as economic consequences, the immediate and longer term costs of providing medical care for abortion-related complications, lost productivity to the country, the impact on families and the community, and the social consequences that affect women and families.

Up to date there is only one previous rigorous RCT with the aim to investigate treatment of incomplete abortion with misoprostol by midwives in comparison to physicians in a low-resource setting.[25] The findings of Klingberg-Allvin et al. (2015), are in coherence with the current result. Therefore, our findings are essential contributions to the scarce evidence that systematically trained midwives in a low-resource setting can independently provide essential elements in PAC. These findings are timely and needed to accomplish standards and guidelines (S&G) regarding CAC and PAC in Kenya and in other African countries lacking such S&Gs.

In conclusion, treatment of incomplete abortion with misoprostol when provided by midwives is as effective, safe, and accepted as when administered by physicians in a low-resource setting. Systematically provided contraceptive counselling composed with a wide range of contraceptive choices in PAC is effective to mitigate unmet need for contraception. In areas with shortages of physicians and scarce resources, midwives would greatly increase women's access to safe and highly accepted PAC.

Data sharing

Additional data is available by emailing marlene.makenzius@ki.se

Declaration of interests

We declare no competing interests.

Funds

The Swedish Research Council (2012-06114; 2011-03369), and the Swedish Research Council on Health, Working Life and Welfare (2015-01398_3).

For peer review only - http://bmjopen.bmj.com/site/about/guidelines.xhtml

Acknowledgements

The authors acknowledge Caroline Nyandat and Sam Owoko for training and providing technical support, the data assistants Christine Apondi and Beatrice Otieno, the staff of Ministry of Health in Kisumu, Dr Paul Mitei, and Prof. Edwin Were, Moi University, who made this project possible and facilitated its implementation. Thanks to Ulla Romild at the National Agency of Public Health, Sweden, for statistical advice. Finally, we thank all the women who participated in our study, and all the providers involved in the study who remained committed and supportive, throughout.

REFERENCES

1. Sedgh G, Bearak J, Singh S, et al. Abortion incidence between 1990 and 2014: global, regional, and subregional levels and trends. *Lancet* 2016; **388**(10041): 258–67.
2. Ziraba AK, Izugbara C, Levandowski BA, et al. Unsafe abortion in Kenya: a cross-sectional study of abortion complication severity and associated factors. *BMC Pregnancy Childbirth* 2015; **15**: 34.
3. Kapp N, Glasier A. WHO technical and policy guidance emphasizes the health systems' responsibility to provide safe abortion services. *Contraception* 2013; **87**(5): 511–2.
4. Singh S. Hospital admissions resulting from unsafe abortion: estimates from 13 developing countries. *Lancet* 2006; **368**(9550): 1887–92.
5. Mayi-Tsonga S, Oksana L, Ndombi I, Diallo T, de Sousa MH, Faundes A. Delay in the provision of adequate care to women who died from abortion-related complications in the principal maternity hospital of Gabon. *Reprod Health Matters* 2009; **17**(34): 65–70.
6. Ganatra B, Tuncalp Ö, Johnston HB, Johnson BR Jr, Gülmezoglu AM, M. T. From concept to measurement: operationalizing WHO's definition of unsafe abortion. *Bull World Health Organ* 2014; **92**: 155.
7. National Council for Law Reporting. Constitution of Kenya 2010 In: Attorney-General NCfLRwtAot, editor. Nairobi; 2010.
8. Johnston HB, Gallo MF, Benson J. Reducing the costs to health systems of unsafe abortion: a comparison of four strategies. *J Fam Plann Reprod Health Care* 2007; **33**(4): 250–7.
9. Vlassoff M, Walker D, Shearer J, Newlands D, Singh S. Estimates of health care system costs of unsafe abortion in Africa and Latin America. *Int Perspect Sex Reprod Health* 2009; **35**(3): 114–21.
10. Prada E, Maddow-Zimet I, Juarez F. The cost of postabortion care and legal abortion in Colombia. *Int Perspect Sex Reprod Health* 2013; **39**(3): 114–23.
11. Kabiru CW, Ushie BA, Mutua MM, Izugbara CO. Previous induced abortion among young women seeking abortion-related care in Kenya: a cross-sectional analysis. *BMC Pregnancy Childbirth* 2016; **16**: 104.
12. Maina BW, Mutua MM, Sidze EM. Factors associated with repeat induced abortion in Kenya. *BMC Public Health* 2015; **15**: 1048.
13. Ochako R, Izugbara C, Okal J, Askew I, Temmerman M. Contraceptive method choice among women in slum and non-slum communities in Nairobi, Kenya. *BMC Womens Health* 2016; **16**: 35.
14. Postabortion Care Consortium. PAC Model. 2015. http://pac-consortium.org/resources/pac-model/ (accessed November 12 2016).
15. Gemzell-Danielsson K, Fiala C, Weeks A. Misoprostol: first-line therapy for incomplete miscarriage in the developing world. *BJOG* 2007; **114**(11): 1337–9.
16. Weeks A, Alia G, Blum J, et al. A randomized trial of misoprostol compared with manual vacuum aspiration for incomplete abortion. *Obstet Gynecol* 2005; **106**(3): 540–7.

For peer review only - http://bmjopen.bmj.com/site/about/guidelines.xhtml

17. Dao B, Blum J, Thieba B, et al. Is misoprostol a safe, effective and acceptable alternative to manual vacuum aspiration for postabortion care? Results from a randomised trial in Burkina Faso, West Africa. *BJOG* 2007; **114**(11): 1368–75.
18. African Population and Health Research Center, Ministry of Health, Kenya, Ipas, and Guttmacher Institute. *Incidence and Complications of Unsafe Abortion in Kenya: Key Findings of a National Study* Nairobi. Nairobi, Kenya, 2012.
19. Berer M. Provision of abortion by mid-level providers: international policy, practice and perspectives. *Bull World Health Organ* 2009; **87**(1): 58–63.
20. Fulton BD, Scheffler RM, Sparkes SP, Auh EY, Vujicic M, Soucat A. Health workforce skill mix and task shifting in low income countries: a review of recent evidence. *Hum Resour Health* 2011; **9**: 1.
21. Osur J, Baird TL, Levandowski BA, Jackson E, Murokora D. Implementation of misoprostol for postabortion care in Kenya and Uganda: a qualitative evaluation. *Glob Health Action* 2013; **6**: 1–11.
22. Renner RM, Brahmi D, Kapp N. Who can provide effective and safe termination of pregnancy care? A systematic review*. *BJOG* 2013; **120**(1): 23–31.
23. Cleeve A, Oguttu M, Ganatra B, et al. Time to act-comprehensive abortion care in east Africa. *Lancet Glob Health* 2016; **4**(9): e601–2.
24. Warriner IK, Wang D, Huong NT, et al. Can midlevel health-care providers administer early medical abortion as safely and effectively as doctors? A randomised controlled equivalence trial in Nepal. *Lancet* 2011; **377**(9772): 1155–61.
25. Klingberg-Allvin M, Cleeve A, Atuhairwe S, et al. Comparison of treatment of incomplete abortion with misoprostol by physicians and midwives at district level in Uganda: a randomised controlled equivalence trial. *Lancet* 2015; **385**(9985): 2392–8.
26. Grimes DA, Benson J, Singh S, et al. Unsafe abortion: the preventable pandemic. *Lancet* 2006; **368**(9550): 1908–19.
27. Shah IH, Ahman E, Ortayli N. Access to safe abortion: progress and challenges since the 1994 International Conference on Population and Development (ICPD). *Contraception* 2014; **90**(6 Suppl): S39–48.
28. Izugbara C, Egesa C, Okelo R. 'High profile health facilities can add to your trouble': Women, stigma and un/safe abortion in Kenya. *Soc Sci & Med* 2015; **141:** 9-18.
29. Turner KL, ed. Medical abortion training guide, 2nd edn. Chapel Hill, NC: Ipas, 2010.29.
30. WHO. Medical eligibility criteria for contraceptive use. 2016. http://www.who.int/reproductivehealth/publications/family_planning/MEC-5/en/ (accessed Feb 02 2013).
31. Piaggio G, Elbourne DR, Pocock SJ, Evans SJ, Altman DG, Group C. Reporting of noninferiority and equivalence randomized trials: extension of the CONSORT 2010 statement. *JAMA* 2012; **308**(24): 2594–604.

For peer review only

For peer review only - http://bmjopen.bmj.com/site/about/guidelines.xhtml

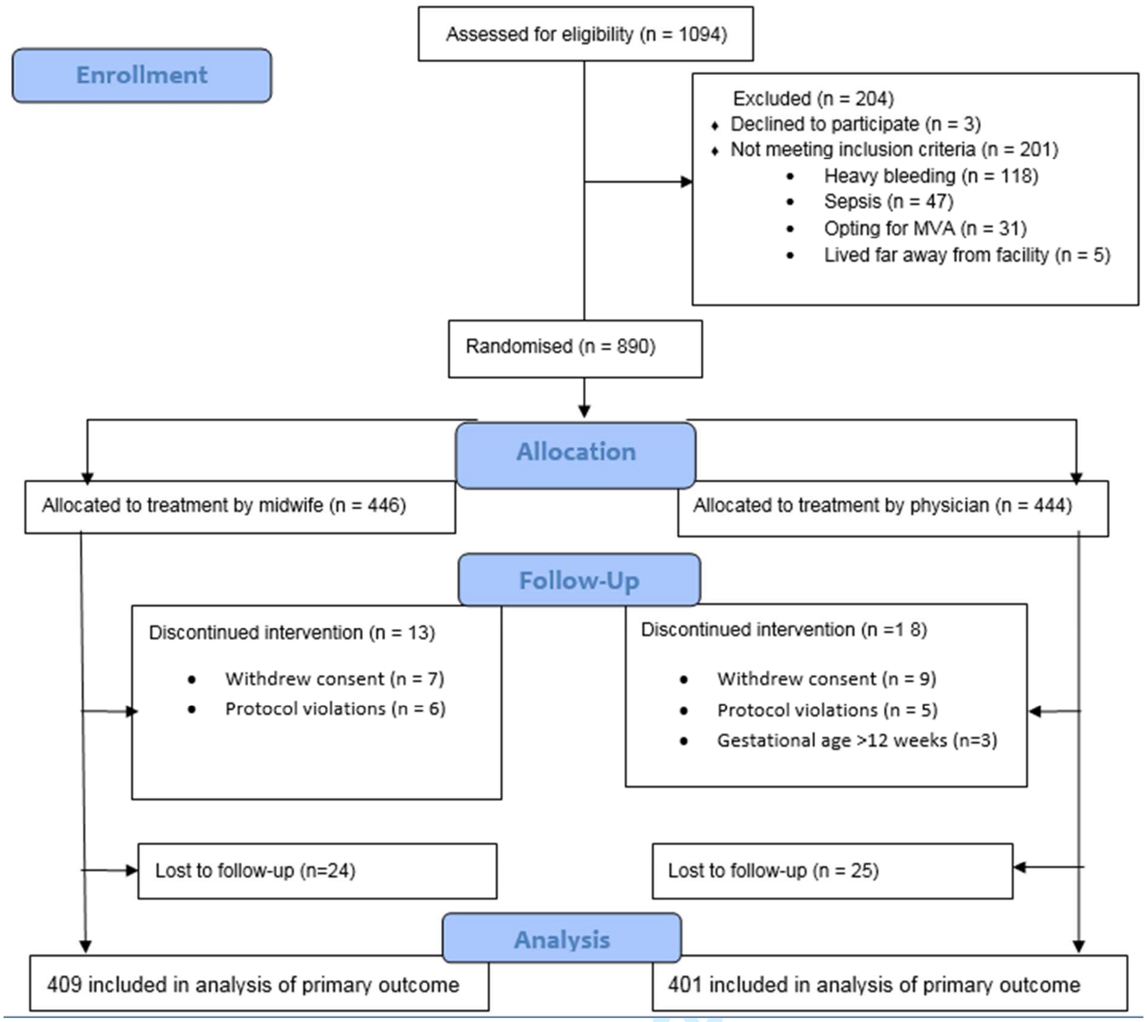

Figure: Trial profile

Table 1: Background characteristics of participating PAC providers.

	**Midwife n = 19 (51.4%)**	**Physician group n =18 (48.6%)**	**Total n = 37 (100%)**
**Age**			
Mean (SD)	47.3 (6.5)	35.9 (10.0)	41.7 (10.1)
Median	50 (6.0)	30 (14.5)	44 (20)
Range	28–55	26–60	26–60
**Sex**			
Female	18 (94.7%)	6 (33.3%)	24 (64.9%)
Male	1 (5.3%)	12 (66.7%)	13 (35.1%)
**Years of professional practice**			
Mean (SD)	22.4 (7.9)	8.8 (8.1)	15.8 (10.5)
Median (IQR)	26 (8.0)	4 (15)	17 (22)
Range	1– 30	1–28	1–30
**Clinical experience in PAC (years)***			

For peer review only - http://bmjopen.bmj.com/site/about/guidelines.xhtml

Mean (SD)	2.7 (1.6)	8.4 (6.9)	5.5 (5.5)
Median (IQR)	3 (3.0)	4 1(4.25)	3 (2.5)
Range	0–5	0–25	0–25

Data are n (%), mean (SD), or median (IQR). *Before study start.

Table 2: Sociodemographic characteristics and reproductive history of participants* by provider

	**Midwife**	**Physician**	**Total**
**Age (years)**			
n	422	416	838
Mean (SD)	25.2 (5.9)	24.8 (5.4)	25.0 (5.6)
Range	14–45	12–41	12–45
14–19	60 (14%)	56 (14%)	116 (14%)
20–24	171 (40%)	167 (40%)	338 (40%)
25–29	101 (24%)	113 (27%)	214 (26%)
30–34	53 (13%)	51 (12%)	104 (12%)
35–39	28 (7%)	26 (6%)	54 (7%)
40–47	9 (2%)	3 (1%)	12 (1%)
**Marital status**			
n	433	426	859
Married or cohabiting	289 (67%)	292 (68%)	581 (68%)
Single	128 (30%)	124 (29%)	252 (29%)
Divorced/separated	9 (2%)	7 (2%)	16 (2%)
Widowed	7 (2%)	3 (1%)	10 (1%)
**Religion**			
n	433	426	859
Christian	427 (99%)	417 (98%)	844 (98%)
Muslim	6 (1%)	9 (2%)	15 (2%)
**Education**			
n	433	425	858
None	6 (1%)	2 (1%)	8 (1%)
Primary 1–4	11 (2%)	9 (2%)	20 (2%)
Primary 1–5	108 (25%)	121 (28%)	229 (27%)
Secondary	206 (48%)	186 (44%)	392 (46%)
Tertiary	102 (24%)	107 (25%)	209 (24%)
**Occupation**			
n	431	423	854
Unemployed	210 (49%)	197 (47%)	407 (48%)
Formal employment	73 (17%)	75 (18%)	148 (17%)
Self-employed	148 (34%)	151 (36%)	299 (35%)
**Gestational age based on clinical examination (weeks)**			
n	433	426	859
Mean (SD)	9.4 (2.2)	9.7 (2.1)	9.6 (2.2)
Range	1–12	3–12	1–12
**Number of previous pregnancies**			
n	433	425	858
Mean (SD)	1.75 (1.8)	1.8 (1.6)	1.8 (1.7)
Range	0–12	0–8	0–12
0	135 (31%)	101 (24%)	236 (27%)
1	94 (22%)	110 (26%)	204 (24%)
2–3	136 (31%)	157 (37%)	293 (34%)
4–5	58 (13%)	50 (11%)	108 (13%)
6–8	7 (2%)	7 (2%)	14 (2%)
9–12	3 (1%)	0	3
**Parity (live birth)**			
n	431	425	856

For peer review only - http://bmjopen.bmj.com/site/about/guidelines.xhtml

Mean (SD)	1.1 (1.4)	1.1 (1.3)	1.1 (1.3)
Range	0-10	0-7	0-10
0	205 (48%)	178 (42%)	383 (45%)
1	105 (24%)	111 (26%)	216 (25%)
2-3	94 (22%)	115 (27%)	209 (24%)
4-5	23 (5%)	18 (4%)	41 (5%)
6-8	3 (1%)	3 (1%)	6 (1%)
9-11	1	0	1

Data are n (%) unless otherwise stated.
*Includes women lost to follow-up.
Internal drop-out range between 0–11 (0–2.6%), among the midwife and physician group, respectively.

Table 3: Outcome of treatment of incomplete abortion by provider

	Midwife	Physician	Risk difference (95% CI)	Adjusted difference (95% CI)*
Randomised and received intervention	446	444		
Per-protocol	409	401		
Complete abortion	390 (95.4%)	378 (94.3%)	1.0% (–4.1 to 2.0)	1.0% (–4.1 to 2.2)

*Adjusted for facility, age (<25 vs ≥25 years), marital status (single [single, widowed, divorced, separated] vs married or cohabiting), education (none or primary 1–8 vs secondary or tertiary), number of previous pregnancies (0 vs >1), and parity (para 0 vs ≥1).

Table 4: Reported secondary outcomes

	**Midwife**	**Physician**	**Total**
**Bleeding since treatment**			
**N**	408	401	806
Less than normal menstrual bleeding	274 (67%)	268 (67%)	542 (67%)
Same as normal menstrual bleeding	78 (19%)	70 (18%)	148 (19%)
Havier than normal menstrual bleeding	56 (14%)	60 (15%)	116 (14%)
**Days bleeding**			
N	396	389	785
Mean	4.2 (2.0)	4.08 (1.7)	4.1 (2.0)
Range	1–14	1–10	1–14
**Pain following treatment**			
N	407	399	806
None	86 (21%)	84 (20%)	170 (21%)
Mild	200 (49%)	194 (49%)	394 (49%)
Moderate	81 (20%)	76 (19%)	157 (20%)
Severe	40 (10%)	45 (11%)	85 (10%)

For peer review only - http://bmjopen.bmj.com/site/about/guidelines.xhtml

**Unscheduled visit**			
N	403	397	800
Yes	20 (5%)	26 (6%)	46 (6%)
Vaginal bleeding or abdominal pain, or both	11 (3%)	18 (4%)	29 (4%)
Chill/fever or dizziness, or both	6 (1%)	6 (1%)	12 (1%)
**Acceptance of the method**			
N	407	398	801
As expected/expected	121 (30%)	130 (32%)	251 (31%)
Worse than expected/expected	29 (7%)	27 (7%)	56 (7%)
Easier than expected/expected	257 (63%)	241 (61%)	498 (62%)
**Felt safe**			
N	400	399	799
Yes	383 (96%)	378 (95%)	779 (97%)
No	17 (4%)	21 (4%)	38 (3%)
**Will recommend the treatment to a friend**			
N	400	399	799
Yes	383 (96%)	378 (95%)	761 (95%)
No	17 (4%)	21 (5%)	38 (5%)
**Received contraceptive counselling**			
N	405	401	806
Yes	395 (98%)	394 (98%)	789 (98%)
No	10 (2%)	7 (2%)	17 (2%)
**Accepted a contraceptive method**			
N	407	401	803
No	104 (26%)	91 (23%)	195 (24%)
Yes	303 (74%)	310 (77%)	613 (76%)
Injectable	118 (39%)	118 (38%)	236 (39%)
Pills	81 (27%)	85 (28%)	166 (27%)
Condoms	75 (25%)	76 (25%)	151 (25%)
Hormonal implants	24 (8%)	21 (7%)	45 (8%)
IUDs	3 (1%)	5 (2%)	8 (1%)
Permanent contraceptives	-	1	1

Data are n (%) unless otherwise stated.
Internal drop-out range between 0–11 (0–2.6%), among the midwife and physician group, respectively.

Appendix 1. Symptoms followed the treatment of incomplete abortion, self-reported by women.

	Midwife	Physician	Total
**Self-reported side effects***			
**N**	409	401	810
None	201 (49%)	206 (51%)	407 (50%)
Abdominal pain	166 (41%)	139 (35%)	305 (38%)
Chills	51 (12%)	46 (12%)	97 (12%)
Nausea	32 (8%)	32 (8%)	64 (8%
Diarrhoea	27 (7%)	22 (6%)	49 (6%)
Vomiting	12 (3%)	17 (4%)	29 (4%)
Foil smelling vaginal or cervical discharge	9 (2%)	10 (2%)	19 (2%)

Data are n (%). *Several options was allowed to obtain.

For peer review only - http://bmjopen.bmj.com/site/about/guidelines.xhtml

BMJ Open

Post-abortion care with misoprostol - equally effective, safe and accepted when administered by midwives compared to physicians: a randomised controlled equivalence trial in a low-resource setting in Kenya

Journal:	*BMJ Open*
Manuscript ID	bmjopen-2017-016157.R1
Article Type:	Research
Date Submitted by the Author:	19-Mar-2017
Complete List of Authors:	Makenzius, Marlene; Karolinska Institutet Department of Public Health Sciences, Global Health (IHCAR)<br>Oguttu, Monica; Kenya Medical Education Trust - KMET<br>klingberg-allvin, marie; Hogskolan Dalarna Akademin Utbildning Halsa och Samhalle<br>Gemzell, Kristina; Karolinska Universitetssjukhuset, Dept. of Women´s and Children´s Health<br>Odero, Theresa; College of Health Sciences, School of Nursing Sciences, University of Nairobi<br>Faxelid, Elisabeth; Karolinska Institutet Department of Public Health Sciences
**Primary Subject Heading**:	Global health
Secondary Subject Heading:	Medical education and training, Obstetrics and gynaecology, Sexual health, Reproductive medicine, Health policy
Keywords:	EDUCATION & TRAINING (see Medical Education & Training), Community gynaecology < GYNAECOLOGY, Reproductive medicine < GYNAECOLOGY, Health policy < HEALTH SERVICES ADMINISTRATION & MANAGEMENT, Maternal medicine < OBSTETRICS

SCHOLARONE™
Manuscripts

For peer review only - http://bmjopen.bmj.com/site/about/guidelines.xhtml

TITLE

Post-abortion care with misoprostol - equally effective, safe and accepted when administered by midwives compared to physicians: a randomised controlled equivalence trial in a low-resource setting in Kenya.

Short title: Post-abortion care with misoprostol: A randomised controlled trial in a low-resource setting, Kenya

AUTHORS

***Marlene Makenzius (MM),** Postdoctor monitoring the RCT at site, PhD, MPH, CNM
Dept. of Public Health Sciences Global Health (IHCAR), Karolinska Institutet
Postal address: Tomtebodavägen 18A, SE-171 77 Stockholm, Sweden
Phone: +46 761131 076, +254 7342 47498
marlene.makenzius@folkhalsomyndigheten.se; marlene.makenzius@ki.se

Monica Oguttu (MO), PI, Director at KMET, PhD, RM, RN
Kenya Medical Education Trust - KMET
Postal address: Tom Mboya Estate, P.O. Box 6805 – 40103, Kisumu, Kenya
+254 701 839 115 or 721 845948

Marie Klingberg-Allvin (MKA), Professor/Pro Vice-Chancellor, PhD, RM, RN
School of Education, Health and Social studies, Dalarna University
Postal address: Dalarna University, SE-791 88 Falun, Sweden and Dept. of Women´s and Children´s Health, Karolinska Institutet

Kristina Gemzell-Danielsson (KMD), Professor, MD, PhD, Chair Div. of Obstetrics and Gynecology
Dept. of Women´s and Children´s Health, Karolinska Institutet
Postal address: WHO-centre, C1:05, Karolinska University Hospital, SE-171 76 Stockholm, Sweden
Phone: +46 8 517- 72128 (or -79539)

Theresa M A Odero (TO), PhD-stud, MSc, RM, RN
College of Health Sciences, School of Nursing Sciences, University of Nairobi
Postal address: P O Box 19676, code 00202 KNH, Nairobi, Kenya

Elisabeth Faxelid (EF), PI, Sweden), Prof., PhD, RM, RN
Dept. of Public Health Sciences Global Health (IHCAR), Karolinska Institutet
Postal address: Tomtebodavägen 18A, SE-171 77 Stockholm, Sweden

*Corresponding author

I Marlene Makenzius The Corresponding Author of this article contained within the original manuscript which includes any diagrams & photographs within and any related or stand alone film submitted (the Contribution") has the right to grant on behalf of all authors and does grant on behalf of all authors, a licence to the BMJ Publishing Group Ltd and its licencees, to permit this Contribution (if accepted) to be published in the BMJ and any other BMJ Group products and to exploit all subsidiary rights, as set out in our licence set out at: http://www.bmj.com/about-bmj/resources-authors/forms-policies-and-checklists/copyright-open-access-and-permission-reuse.
Please tick one or more boxes as appropriate:
YES, I am one author signing on behalf of all co-owners of the Contribution.

For peer review only - http://bmjopen.bmj.com/site/about/guidelines.xhtml

Author's contribution

The lead author for this manuscript was Marlene Makenzius

Study design and planning: Kristina Gemzell Danielsson, Elisabeth Faxelid, Marie Klingberg Allvin and Monica Oguttu.
Training and implementation of the study: Marie Klingberg Allvin, Monica Oguttu, Kristina Gemzell Danielsson, Elisabeth Faxelid, Theresa Odero and Marlene Makenzius.
Data Collection: Marlene Makenzius, Monica Oguttu and Theresa Odero
Data analysis: Marlene Makenzius, cross-checked by a senior statistician, Ulla Romild, at the National Agency of Public Health in Sweden.
Data interpretation: Marlene Makenzius, Elisabeth Faxelid, Kristina Gemzell Danielsson, Marie Klingberg Allvin, Monica Oguttu and Theresa Odero.
Literature search: Marlene Makenzius
Writing: Marlene Makenzius
Editing and proof-reading: Kristina Gemzell Danielsson, Elisabeth Faxelid, Marie Klingberg Allvin, Monica Oguttu, Theresa Odero, and Scribendi (language editing).

ABSTRACT

Objective To assess the effectiveness of midwives administering misoprostol to women with incomplete abortion seeking post-abortion care (PAC), compared with physicians.

Design A multicentre randomised controlled equivalence trial. The study was not masked.

Settings Gynaecological departments in two hospitals in a low-resource setting, Kenya.

Population Women (n=1094) with incomplete abortion in the first trimester, seeking PAC during 1 June 2013 to 30 August. Participants were randomly assigned to receive treatment from midwives or physicians. 409 and 401 women in the midwife and physician groups, respectively, were included in the per-protocol analysis.

Interventions 600µg misoprostol orally, and contraceptive counselling by a physician or midwife.

Main outcome measures Complete abortion not needing surgical intervention within 7–10 days. The main outcome was analysed on the per-protocol population with a generalised estimating equation model. The predefined equivalence range was –4 % to 4%. Secondary outcomes were analysed descriptively.

Results The proportion of complete abortion was 94.8% (768/810), 390 (95.4%) in the midwife-group, and 378 (94.3%) in the physician-group. The proportion of Incomplete abortion was 5.2% (42/810), similarly distributed between midwives and physicians. The model-based risk difference for midwives versus physicians was 1.0% (–4.1 to 2.2). Most women felt safe (97%; 779/799), 93% (748/801) perceived the treatment as expected/easier than expected. After contraceptive counselling the uptake of a contraceptive method after 7-10 days occurred in 76% (613/810). No serious adverse events were recorded.

Conclusions Treatment of incomplete abortion with misoprostol provided by midwives is equally effective, safe, and accepted by women as when administered by physicians in a low-resource setting. Systematically provided contraceptive counselling in PAC is effective to mitigate unmet need for contraception.

The trial was registered at ClinicalTrials.gov, NCT01865136

For peer review only - http://bmjopen.bmj.com/site/about/guidelines.xhtml

Strengths

- Despite a restrictive abortion law the follow-up rate was considered high.
- A rigorous project design and trial implementation was adopted in collaboration with Ministry of Health, different cadres of health care providers, leaders, NGOs, and universities.

Limitations

- District hospitals are usually more advanced regarding staffing and resources and might therefore not be representative of the small health dispensaries located in the most remote areas of the region and other parts of the country.
- The misoprostol-treatment was highly accepted among the treated women, however the measurement was based on retrospective self-reported statements.
- The contraceptive up-take was high at follow-up (7-10 days), but lacks long-term follow-up.

INTRODUCTION

Since 1990, the annual abortion rate has declined in high-income countries, yet remains the same in low-income countries.[1] Consequently, the estimated annual abortion rate is now higher in low-income countries (37 per 1,000 women) than in high-income countries (27 per 1,000 women).[1]

Although induced abortion is medically safe when done in accordance with recommended guidelines, many women undergo unsafe procedures that put their health and life at risk.[2-5] Unsafe abortion is defined by WHO as a procedure performed under conditions that fail to meet the minimal medical standards and/or by untrained persons.[6] The majority of unsafe abortions occur in low-income countries where induced abortion is restricted, and is a leading cause of death among young women in sub-Saharan Africa.[1,2,4,5] Under the 2010 Kenyan constitution, abortions may be induced if the woman needs emergency treatment or her life or health is in danger.[7]

In Kenya the total fertility rate was 4.6 in 2008-09, contraceptive prevalence was 46%, and 42% of married women reported their current pregnancies as unintended.[2] Induced abortion in 2012 was estimated to occur in 48 in 1000 women of reproductive age (15–49 years).[2] Even though some of these abortions were safe and legally done in public and private facilities, most abortions were done outside authorized facilities and thus considered unsafe.[2]

The costs for post-abortion care (PAC) constitute a significant financial burden on public healthcare systems.[4,8-10] It is estimated that seven million women in low-income countries are treated for complications from unsafe abortions every year and at least 22,000 die from abortion-related complications every year.[1] In Kenya, an estimate of 120,000 women are treated for complications resulting from unsafe abortion annually[2], and 35% of maternal deaths are attributed to unsafe abortions.[2] The proportion of severe complications such as high fever, sepsis, shock, and organ failure is high (37%), and associated factors are delay in seeking care and interference with the

For peer review only - http://bmjopen.bmj.com/site/about/guidelines.xhtml

pregnancy.[2,11] About 71% of women seeking PAC did not use a contraceptive method prior to becoming pregnant.[2]

PAC is an integral component of comprehensive abortion care (CAC), and is often used in settings where abortion is legally restricted.[12] Essential elements are community and service provider partnerships, counselling, treatment of incomplete and unsafe abortion, contraceptive services, and linkages to reproductive and other health services.[12] The prostaglandin E_1 analogue misoprostol is an effective drug to treat incomplete abortion in the first trimester, in PAC[13], and no significant differences has been reported in the effectiveness between misoprostol and manual vacuum aspiration (MVA) for the treatment of incomplete abortion.[14,15] Misoprostol simplifies the procedure, compared to MVA which is more intrusive.[13]

The use of MVA and medical abortion is growing in Kenya; however, other less safe procedures remain common.[16] There is also a mismatch between where maternal mortality exists and where health workers are, suggesting critical inequities in Kenya in the availability for health care following international standards and guidelines.[16] In addition, nurses and midwives tend to have a subordinate role compared to physicians and have traditionally been excluded from decision-making.

Shifting/sharing tasks may increase access to and availability of services without compromising performance or patient outcomes and may be cost-effective.[17-21] Renner et al. (2013) found limited evidence indicating that trained mid-level providers may effectively and safely provide first trimester termination of pregnancy.[20] Data are limited by the scarcity of RCTs and biases of the cohort studies.[20.] However, trained nurses and midwives have in some RCTs been proven to provide safe medical abortions[22,23] and to treat incomplete abortion as effective as physicians.[24] Yet, regulations restrict misoprostol prescription and supervision to physicians, and the high proportion of complications and high fatality rate indicate that unsafe abortions continue to pose a major public health challenge in low-income settings.[1,2, 25]

Though it is an urgent need to provide timely and effective treatments at health facilities, ensuring the availability of medical equipment, personnel, knowledge and skills, is a tremendous challenge in resource-limited economies.[18,19,25] This hamper women’s access to proper care, and forces them to obtain unsafe abortions.[18,19,26,27]

The overarching aim with this trial was to assess the effectiveness of midwives administering misoprostol to women with incomplete abortion seeking post-abortion care, compared with physicians.

For peer review only - http://bmjopen.bmj.com/site/about/guidelines.xhtml

METHODS

The study was a multicentre randomised parallel-group, and the study protocol and trial followed the CONSORT guidelines for non-inferiority and equivalence randomised trials.[28]

The project was conducted at the Jaramogi Oginga Odinga Teaching and Referral Hospital (JOOTRH) and Kisumu County Hospital (KCH) in Kisumu County, western Kenya. Regional variations in abortion incidence exist in Kenya, and the rate in this region (Nyanza/Western) was among the highest, 63 induced abortions per 1000 women of reproductive age (15-49 years), compared with 48 for the country. [18] Kisumu is the third largest city in Kenya and has a population of 500,000. The project was implemented in the gynaecological ward within the Department of Obstetrics and Gynaecology at the two facilities (June 1th, 2013 until August 31th 2016). Altogether the two facilities admit around 26 women with incomplete abortions per month. Midwives (n = 19) and physicians (n = 18) included in the project, Table 1, underwent a standardised training.[29,30]

The healthcare facilities selected for inclusion were equipped by the project to provide basic and emergency obstetric services and were estimated to have sufficient staff and resources required for the trial. The procedure and the instruments were pilot tested at the two hospitals before the study started. The first two months was set to a run-in phase, in which the facilities were given time to set the routine among staff for screening, enrolment, and follow-up, as well as to emancipate space to store a large number of protocol-records safely, and to provide counselling in a secluded room. Minor revisions were made to the instruments and the recruitment procedure.

Patient involvement

The study was designed and coordinated by researchers at the Department of Public Health Sciences, Global Health (IHCAR), and Women's and Children's Health, Karolinska Institutet, Stockholm, Sweden; Kisumu Medical Education Trust (KMET); University of Nairobi; and Moi University, Eldoret, Kenya; and at JOOTRH and KCH in Kisumu, Kenya.

Research question and outcome measures was constructed by the researcher, and health care providers at the study facilities who were experienced in PAC, in order to strengthen the validity. The preliminary results was continuously reported during the study period to the two facilities by the study coordinator to encourage the provider's work, who also used the information to encourage the participating women. The outcome and acceptability of the intervention was evaluated by the provider at follow-up, and the women were advised to seek care ahead of the appointment if they obtained unexpected symptoms/burden.

For peer review only - http://bmjopen.bmj.com/site/about/guidelines.xhtml

We obtained ethical approval from the JOOTRH Ethical Review Committee (diary number ERC42/13), and the Swedish regional Ethics Committee in Stockholm (2013/902-31/1). All the patients gave written informed consent. The participants could withdraw from participation at any time without consequences. Consent forms were translated into the local languages Kiswahili and Luo and back translated into English to ensure accurate translation. The form was read to each participant and those unable to write marked the form with a thumb print, and a witness signed to indicate that the study had been explained to the participant and that she understood and accepted to participate.

Inclusion and exclusion criteria

Inclusion criteria were women with signs of incomplete abortion, based on clinical assessments (i.e., positive pregnancy test, bleeding, contractions and lower abdominal pain, open cervical os, and partial expulsion) and physical examination (including vaginal examination), but without the use of ultrasound. Exclusion criteria were complete abortion (no bleeding or contractions), , a uterine size estimated over 12 weeks of gestation, suspected ectopic pregnancy (severe abdominal pain), unstable haemodynamic status, signs of pelvic infection or sepsis, and known allergy to misoprostol.

Randomisation and masking

Eligible women who consented to participate were randomly allocated to a midwife (intervention) or standard care with a physician (control) for diagnosis and treatment. The randomisation was done in blocks of eight and was stratified for study site. We used a computer random number generator to generate a list of codes from 1 to 890 and each code was linked to one of the two study groups. Sequentially numbered, opaque, sealed envelopes, each containing a random allocation, were prepared at the coordinating department of KMET, and later opened in consecutive order by the research assistants after obtaining written consent. Data management was organised locally at the coordinating department at KMET, Kisumu.

The study coordinator checked protocols for accuracy, corrected protocols after discussion with research assistants, and did continuous process evaluation. Study protocols were collected and data was entered continuously throughout data collection. The providers received support and guidance throughout the study period by the study coordinator. The study was not masked to the study participants or providers.

Procedures

Eligible providers for participation were midwives and physicians involved in PAC at the two facilities in Kisumu, where the superior regime to treat first trimester incomplete abortion was MVA (62%),

For peer review only - http://bmjopen.bmj.com/site/about/guidelines.xhtml

For peer review only

similar to the country (65%. Medical abortion catered for only 11% (27% other methods).[18] In the region about 37% of women (15-49) had ever used a contraceptive method compared to 46% in the country.[18] The healthcare providers were trained in a 5-day training program according to Kenyan and the WHO standardised PAC training module.[28,29] The training was both theoretical and practical and ongoing facility-based continuous medical education occurred throughout the project period in order to cover all/new staff. This training included knowledge on diagnosing incomplete abortions, treatment with misoprostol and MVA, and post-abortion contraceptive counselling as well as interview technique.

Four midwives per facility were trained as research assistants in the study and were responsible to oversee and monitor supply in stock (misoprostol and contraceptives such as condoms, pills, Depo-Provera, hormonal implants and intra uterine devices [IUD]. In addition, the research assistants were responsible for the screening, the follow-up visit including assessment of abortion status at follow-up and contraceptive uptake, and performing MVA when necessary. Women admitted with signs of incomplete abortion were first screened based on self-reported last menstrual period, pregnancy test and symptoms. Eligible women who consented to participate were randomly allocated to the midwife or physician group. The clinical assessment included (Table 2); the last menstrual period, obstetric and gynaecological history, pelvic examination including any signs of genital infections, cervical status, bleeding, and size of the uterus. Ultrasound was not used.

Each participant was given one single dose of 600 µg misoprostol orally. Additionally, they were offered analgesics (ibuprofen or paracetamol) and oral antibiotics given according to local guidelines for PAC. For monitoring purposes, participants were advised to stay at the clinic for 4 to 12 hours after swallowing the misoprostol tablets. Before discharge, all women were offered contraceptive counselling and provided with a contraceptive method if they wanted, and given a follow-up appointment after 7–10 days.

Participants received detailed information about bleeding, pain expected, abnormal symptoms after treatment (severe pain, fever, and foul smelling vaginal discharge), and were informed about the importance of seeking care or consulting on the toll-free line open 24/7 if such symptoms occurred. Side-effects were recorded (Appendix 1). The women were offered reimbursement for travel costs as an encouragement to come for follow-up. All women received an SMS reminder two days before the scheduled follow-up appointment. Women who did not show up were contacted by the research assistant using a cell phone provided by the project, and the woman was given a new appointment and encouraged to attend.

For peer review only - http://bmjopen.bmj.com/site/about/guidelines.xhtml

Outcomes

The pre-specified primary outcome was complete abortion, requiring no further medical or surgical intervention, Table 3. A symptom diary card was used to assess outcomes following the treatment (Table 4)

(i) daily bleeding in relation to normal menstrual bleeding,
(ii) pain following the treatment,
(iii) unscheduled visits at a healthcare facility and the reason,

The following secondary outcomes were assessed(Table 5):

(iv) women's acceptability of the treatment was recorded by three questions "How did you perceive the treatment procedure", "Did you feel safe after the treatment", and will you recommend the treatment to a friend or a relative", and
(v) contraceptive uptake was recorded by three questions "Did you receive contraceptive counselling, "Did you accept a contraceptive method", "What method did you chose" (Table 5).

Statistical analyses

The sample size was calculated with the objective of showing two-sided equivalence assuming that the rate of incomplete abortions could be 4% and would apply to both types of providers.[24,27] A pre-defined acceptable difference in completion rate between the two providers ranged from –4% to +4%. To establish equivalence with a power of 80% and two-sided 95% CI, the required sample size was 400 per group. Compensation for about 10% loss to follow-up gave a total sample size of 888. Background characteristics and secondary outcomes are presented with descriptive statistics, as per the pre-protocol.

Analysis of the primary outcome (complete abortion) was made by per-protocol analysis with a generalised estimating equation model to estimate the risk difference between the two providers with (two-sided) 95% CI. The intention-to-treat population was considered to be all randomised women with data for the primary outcome. If the CI of the risk difference between the two groups fell within the predetermined margin of equivalence (-4% to +4%), the two types of providers were considered equivalent. P values of 0.05 or less were considered statistically significant. Additionally, we adjusted for the following covariates: facility, age (<25 vs ≥25 years), marital status (single vs married or cohabiting), education (none, primary 1–4, or 5–8 vs secondary or tertiary), number of previous pregnancies (1 vs >1), and live births (0 vs ≥1). We estimated the adjusted risk difference as the predicted risk difference at the average of all included covariates. Equivalence between the two study groups can be stated if the 95% CI of the risk difference lies completely within the predetermined limits of equivalence (–4% to 4%). Safety parameters of the procedure were viewed

For peer review only - http://bmjopen.bmj.com/site/about/guidelines.xhtml
For peer review only

descriptively without any formal statistical testing. The study protocol was not changed after the run-in period. All data were entered and cleaned in IBM SPSS Statistics for windows version 22.0, and we did all the statistical analysis within this software. The trial is registered at ClinicalTrials.gov, number NCT01865136.

Role of the funding source

The authors designed this an investigator-initiated trial, in collaboration with healthcare providers and management at the study setting. The funders did not play any role in the study design or in the collection, analysis, and interpretation of data. All authors had full access to the data, and the first author regularly reported preliminary results to the whole research team. The corresponding author had final responsibility for the decision to submit the manuscript for publication.

RESULTS

From 1 June 2013 to 31 May 2016, we assessed 1094 women with signs of incomplete abortion for eligibility for participation in this study. Of these, 201 were ineligible, and 3 declined to participate. We randomly assigned 890 women to the midwife group (n = 446) and physician group (n = 444). A total of 31 women were excluded, of which 13 had been allocated to the midwife group (7 withdrew consent at follow-up and 6 were classified as protocol violations) and 18 had been allocated to the physician group (9 withdrew consent at follow-up, 5 were classified as protocol violations, and 3 had a gestational age of >12 weeks). A total of 49 women were lost to follow-up (24 in the midwife group and 25 in the physician group; Figure 1). Finally, a total of 810 women, 409 in the midwife group and 401 in the physician group, received misoprostol treatment with misoprostol and were included in the per-protocol analysis.

Among the providers, the midwives had longer work experience than the physicians; on the other hand, the physicians were more experienced in PAC than the midwives in PAC (Table 1).

The baseline characteristics such as sociodemographic background and reproductive history were similarly distributed between the two groups (Table 1). The mean duration of gestational age based on clinical examination was 9.6 weeks (range 1–12 weeks; Table 2).

Sub-analyses showed slightly higher proportions of young (< 25 years), single, unemployed, and nulliparous women among those who dropped out of the study compared to those who came for the follow-up visits. There were no differences in gestational age or education levels between the two groups. None of the results of the sub-analyses were statistically significant (p > 0.05).

Main outcomes

For peer review only - http://bmjopen.bmj.com/site/about/guidelines.xhtml

The overall proportion of complete abortion was 94.8% (768/810). As shown in Table 3, 390/409 (95.4%) women in the midwife group and 378/401 (94.3%) women in the physician group had complete abortions (Table 3). The model-based risk difference for midwife versus physician group was 1.0% (–4.1 to 2.0). There were 42/810 incomplete abortions (5.2%), 19/409 (4.6%) in the midwife group and 23/401 (5.7%) in the physician group. Among the 42 women with incomplete abortions, 25 were treated with MVA and 17 were treated with a repeat dosage of misoprostol after they completed the follow-up assessment. Among the 25 women who obtained MVA, 2 were treated with a repeat dosage of misoprostol prior to MVA. No serious adverse events were recorded.

Most women (690; 86%) reported bleeding less than or the same as normal menstrual bleeding after treatment, and the mean number of bleeding days was 4.1 (SD 2.0; range, 1–14). A majority (564/806; 70%) of the women reported no or mild pain after treatment, and 6% (46/806) women reported unscheduled visits. Vaginal bleeding and abdominal pain were reported as reasons for the unscheduled visits (Table 4). There were no differences in the secondary outcomes between the groups. 407/810 women reported none symptoms following the treatment, while 402/810 women reported symptoms; abdominal pain, chills, nausea, diarrhoea, vomiting, foul smelling vaginal and/or cervical discharge, and were similar in both groups (Appendix 1).

Secondary outcomes

Table 4 shows that most women perceived the treatment to be as expected or easier than expected (748/801; 93%), and 97% felt safe during the procedure (779/799). Additionally, 95% (761/799) women stated that they would recommend the treatment to a friend.

Contraceptive counselling was provided to 789 of the 810 (98%) women; of these, 613 (76%) accepted a contraceptive method of their own choice (Table 5). The following choices of contraceptive methods were offered, and distributed as follows: injectable (236/613; 39%), pills (166/613; 27%), condoms (151/613; 25%), hormonal implant (45/613; 8%), IUD (8/613; 1%), and permanent contraception (1/613; 0.16%).

DISCUSSION

Our finding shows that treating women with incomplete abortion (≤12 weeks of gestation), with misoprostol was found to be equally effective and safe when provided by midwives or physicians in a low-resource setting in Kenya. In addition, the treatment is highly accepted among the treated women (93%), and the uptake of a contraceptive method is high (76%) after 7-10 days when contraceptive counselling is systematically provided in post-abortion care (PAC).

For peer review only - http://bmjopen.bmj.com/site/about/guidelines.xhtml

The study design strengthen the reliability and the validity by the sample size, high follow-up rate and the socio-demographic characteristics of the women (i.e. heterogeneous, though overall low socioeconomics similar to those of the population in the country[2]). There are however weaknesses of the study to be considered. First, the midwives had more years of professional experience than the physicians, but the physicians were more experienced in PAC. No more information was given in detail about their working experience, however, all the providers within the two facilities received the same training in the treatment of incomplete abortion. Secondly, although the socioeconomic and demographic characteristics of study participants were representative of women in fertile ages in Kisumu County, they might not be representative of women living in the most remote rural areas of this county. Third, district hospitals are usually more advanced regarding staffing and resources and might therefore not be representative of the small health dispensaries located in remote areas of the region. Fourth, both types of providers worked in the same hospital and in the same environment. We cannot rule out interactions between the two provider groups, which might have led to a convergence of outcome. Fifth, drop-outs to follow-up was a challenge, as well as the lack of long-term follow up for the contraceptive up-take.

Though we encouraged women to come for follow-up by paying for the local transport, sent SMS reminders ahead of the scheduled appointment, and called the patient if they did not show up, 49 of the 859 women did not come for the follow-up. Some of the women did not have a phone and were thus not reachable, and some of the women who were contacted by phone did not find it necessary to attend the follow-up, as they considered themselves to have recovered without any side effects. Loss to follow-up could also be related to abortion stigma, which is present within the current county and other settings where abortion is restricted.[19,26] Women seeking PAC have previously reported shame and guilt, and they don’t want to be associated with the abortion clinic and its activities once the abortion issue is completed.[19,26] Therefore, in coherence with Osur et al.[19] (2013), instead of considering drop-outs criteria as bias, this might rather be an important reflection of the stigma surrounding abortion.

The overall complete abortion rate was 94.8%, which is comparable with the results from other studies that have used the same regimen (94.5–96.7%)[14,15,22-24] In our study, ultrasonography was not available to any provider, emphasizes that the safety of using misoprostol for treatment of incomplete abortion, in low-resource settings, which can be performed by trained midwives without the routine use of ultrasonography before or after abortion.

Of the women incomplete abortion (42/810), 25 were treated with MVA and 17, with repeated dosage of misoprostol, similarly distributed between the two provider types. Repeat dosage of

For peer review only - http://bmjopen.bmj.com/site/about/guidelines.xhtml

misoprostol is apparently an alternative for MVA when the first dosage of misoprostol has failed. This could be compared with results from a similar study from Uganda where all women with incomplete abortion were treated with MVA (36/955).[24]

A notable additional finding was the frequency of women with sepsis, which was a criterion for ineligibility in this study (47/1094). This proportion is higher, that has been seen in the neighbor country Uganda[2] This might be an important reflection of the magnitude of unsafe abortion and the immense poor state of reproductive health access for women in Kenya. There is no doubt that there is room for improving timely and accessible CAC services, in particular PAC, in Kenya.

Although these results clearly show that misoprostol is a simple, efficient and desirable method, which is highly accepted by women, implementation of PAC in Kenya is hampered by the lack of national clinical guidelines.[16,21] Laws and policies governing access to safe abortion in Africa vary, ranging from very restrictive, such as in Kenya, where abortion is permitted only to save the life of the mother, to liberal, like in South Africa, where abortion is permitted through the 12 week of pregnancy or later.[1] Stigmatizing attitudes and beliefs surrounding abortions in the society[19,26], not least among healthcare providers[26], contribute to women's delay in seeking timely care for abortion-related complications.

Kenya has a policy framework to prevent maternal mortality. However, this policy framework excludes unsafe abortion, which is addressed through the provision of PAC.[7,16,21] In 2012, the Ministry of Health (MH) developed clinical guidelines on prevention of morbidity and mortality from unsafe abortions in Kenya, but the document was arbitrarily withdrawn in 2013. [16,21] A task force was commissioned by the Director of Medical Services to consult and revise the guidelines, and the work was finalised in September 2015, but since then, signing of the document has stalled. Simultaneously, women seeking treatment for complications arising from unsafe abortions is on the rise.[1,16]

New guidelines may not only contribute to increase the quality and effectiveness to treat complications of unsafe abortion, it may also encourage the prevention of unintended pregnancy.[1,16] The current result shows that systematically provided contraceptive counselling to PAC-seeking women is feasible and effective, as three out of four women accepted a contraceptive method. However, the use of modern LARC methods, such as hormonal implants and IUDs, was extremely low, in comparison to injectables. This indicates a pronounced need for concerted efforts to urgently reach women who have an unmet contraceptive need by giving them access to post-abortion contraceptive counselling and modern methods. Mitigating the unmet need for contraception could

For peer review only - http://bmjopen.bmj.com/site/about/guidelines.xhtml

be the key to reducing numbers of subsequent unintended pregnancies and unsafe abortions, as well as maternal morbidity and mortality.

Future research needs to address long-term follow-up on contraceptive adherence after PAC. In spite of the convincing rationality in PAC of preventing future unplanned pregnancies by providing post-abortion contraceptive services, this component remains one of the weakest parts of post-abortion care.[1,2,12] Unsafe abortion has also significant consequences that are less widely recognized, such as economic consequences, the immediate and longer term costs of providing medical care for abortion-related complications, lost productivity to the country, the impact on families and the community, and the social consequences that affect women and families.

In conclusion, treatment of incomplete abortion with misoprostol when provided by midwives is equally effective, safe, and accepted as when administered by physicians in a low-resource setting. Systematically provided contraceptive counselling composed with a wide range of contraceptive choices in PAC is effective to mitigate unmet need for contraception. In areas with shortages of physicians and scarce resources, midwives would greatly increase women's access to safe and highly accepted PAC. These findings are timely and needed to accomplish standards and guidelines (S&G) regarding CAC and PAC in Kenya and in other African countries lacking such S&Gs.

Data sharing

Additional data is available by emailing marlene.makenzius@ki.se

Declaration of interests

We declare no competing interests.

Funds

The Swedish Research Council (2012-06114; 2011-03369), and the Swedish Research Council on Health, Working Life and Welfare (2015-01398_3).

Acknowledgements

The authors acknowledge Caroline Nyandat and Sam Owoko for training and providing technical support, the data assistants Christine Apondi and Beatrice Otieno, the staff of Ministry of Health in Kisumu, Dr Paul Mitei, and Prof. Edwin Were, Moi University, who made this project possible and facilitated its implementation. Thanks to Ulla Romild at the National Agency of Public Health, Sweden, for statistical advice. Finally, we thank all the women who participated in our study, and all the providers involved in the study who remained committed and supportive, throughout.

For peer review only - http://bmjopen.bmj.com/site/about/guidelines.xhtml

REFERENCES

1. Sedgh G, Bearak J, Singh S, et al. Abortion incidence between 1990 and 2014: global, regional, and subregional levels and trends. *Lancet* 2016; **388**(10041): 258–67.
2. Ziraba AK, Izugbara C, Levandowski BA, et al. Unsafe abortion in Kenya: a cross-sectional study of abortion complication severity and associated factors. *BMC Pregnancy Childbirth* 2015; **15**: 34.
3. Kapp N, Glasier A. WHO technical and policy guidance emphasizes the health systems' responsibility to provide safe abortion services. *Contraception* 2013; **87**(5): 511–2.
4. Singh S. Hospital admissions resulting from unsafe abortion: estimates from 13 developing countries. *Lancet* 2006; **368**(9550): 1887–92.
5. Mayi-Tsonga S, Oksana L, Ndombi I, Diallo T, de Sousa MH, Faundes A. Delay in the provision of adequate care to women who died from abortion-related complications in the principal maternity hospital of Gabon. *Reprod Health Matters* 2009; **17**(34): 65–70.
6. Ganatra B, Tuncalp Ö, Johnston HB, Johnson BR Jr, Gülmezoglu AM, M. T. From concept to measurement: operationalizing WHO's definition of unsafe abortion. *Bull World Health Organ* 2014; **92**: 155.
7. National Council for Law Reporting. Constitution of Kenya 2010 In: Attorney-General NCfLRwtAot, editor. Nairobi; 2010.
8. Johnston HB, Gallo MF, Benson J. Reducing the costs to health systems of unsafe abortion: a comparison of four strategies. *J Fam Plann Reprod Health Care* 2007; **33**(4): 250–7.
9. Vlassoff M, Walker D, Shearer J, Newlands D, Singh S. Estimates of health care system costs of unsafe abortion in Africa and Latin America. *Int Perspect Sex Reprod Health* 2009; **35**(3): 114–21.
10. Prada E, Maddow-Zimet I, Juarez F. The cost of postabortion care and legal abortion in Colombia. *Int Perspect Sex Reprod Health* 2013; **39**(3): 114–23.
11. Kabiru CW, Ushie BA, Mutua MM, Izugbara CO. Previous induced abortion among young women seeking abortion-related care in Kenya: a cross-sectional analysis. *BMC Pregnancy Childbirth* 2016; **16**: 104.
12. Postabortion Care Consortium. PAC Model. 2015. http://pac-consortium.org/resources/pac-model/ (accessed November 12 2016).
13. Gemzell-Danielsson K, Fiala C, Weeks A. Misoprostol: first-line therapy for incomplete miscarriage in the developing world. *BJOG* 2007; **114**(11): 1337–9.
14. Weeks A, Alia G, Blum J, et al. A randomized trial of misoprostol compared with manual vacuum aspiration for incomplete abortion. *Obstet Gynecol* 2005; **106**(3): 540–7.
15. Blandine T, Ouattara AZ, Coral A, Hassane C, Clotaire H, Dao B, Lankoande J, Diop A, Blum J. Sublingual [corrected] misoprostol as first-line care for incomplete abortion in Burkina Faso. J.*Int J Gynaecol Obstet.* 2012;**119**(2):166-9.
16. African Population and Health Research Center, Ministry of Health, Kenya, Ipas, and Guttmacher Institute. *Incidence and Complications of Unsafe Abortion in Kenya: Key Findings of a National Study* Nairobi. Nairobi, Kenya, 2012.
17. Huber D, Curtis C, Irani L, Pappa S, Arrington L. Postabortion Care: 20 Years of Strong Evidence on Emergency Treatment, Family Planning, and Other Programming Components. *Glob Health Sci Pract.* 2016; **29**(3):481-94.
18. Fulton BD, Scheffler RM, Sparkes SP, Auh EY, Vujicic M, Soucat A. Health workforce skill mix and task shifting in low income countries: a review of recent evidence. *Hum Resour Health* 2011; **9**: 1.
19. Osur J, Baird TL, Levandowski BA, Jackson E, Murokora D. Implementation of misoprostol for postabortion care in Kenya and Uganda: a qualitative evaluation. *Glob Health Action* 2013; **6**: 1–11.

For peer review only - http://bmjopen.bmj.com/site/about/guidelines.xhtml

20. Renner RM, Brahmi D, Kapp N. Who can provide effective and safe termination of pregnancy care? A systematic review*. *BJOG* 2013; **120**(1): 23–31.
21. Cleeve A, Oguttu M, Ganatra B, et al. Time to act-comprehensive abortion care in east Africa. *Lancet Glob Health* 2016; **4**(9): e601–2.
22. Warriner IK, Wang D, Huong NT, et al. Can midlevel health-care providers administer early medical abortion as safely and effectively as doctors? A randomised controlled equivalence trial in Nepal. *Lancet* 2011; **377**(9772): 1155–61.
23. Olavarrieta CD, Ganatra B, Sorhaindo A, Karver TS, Seuc A, Villalobos A , García SG, Pérez M, Bousieguez M, Sanhueza P. Nurse versus physician-provision of early medical abortion in Mexico: a randomized controlled non-inferiority trial. *Bulletin of the World Health Organization* 2015;93:249-258
24. Klingberg-Allvin M, Cleeve A, Atuhairwe S, et al. Comparison of treatment of incomplete abortion with misoprostol by physicians and midwives at district level in Uganda: a randomised controlled equivalence trial. *Lancet* 2015; **385**(9985): 2392–8.
25. Grimes DA, Benson J, Singh S, et al. Unsafe abortion: the preventable pandemic. *Lancet* 2006; **368**(9550): 1908–19.
26. Izugbara C, Egesa C, Okelo R. 'High profile health facilities can add to your trouble': Women, stigma and un/safe abortion in Kenya. *Soc Sci & Med* 2015; **141:** 9-18.
27. Shah IH, Ahman E, Ortayli N. Access to safe abortion: progress and challenges since the 1994 International Conference on Population and Development (ICPD). *Contraception* 2014; **90**(6 Suppl): S39–48.
28. Piaggio G, Elbourne DR, Pocock SJ, Evans SJ, Altman DG, Group C. Reporting of noninferiority and equivalence randomized trials: extension of the CONSORT 2010 statement. *JAMA* 2012; **308**(24): 2594–604.
29. Turner KL, ed. Medical abortion training guide, 2nd edn. Chapel Hill, NC: Ipas, 2010.29.
30 WHO. Medical eligibility criteria for contraceptive use. 2016. http://www.who.int/reproductivehealth/publications/family_planning/MEC-5/en/ (accessed Feb 02 2013).

Table 1: Background characteristics of participating PAC providers.

	**Midwife n = 19 (51.4%)**	**Physician group n =18 (48.6%)**	**Total n = 37 (100%)**
**Age**			
Mean (SD)	47.3 (6.5)	35.9 (10.0)	41.7 (10.1)
Median	50 (6.0)	30 (14.5)	44 (20)
Range	28–55	26–60	26–60
**Sex**			
Female	18 (94.7%)	6 (33.3%)	24 (64.9%)
Male	1 (5.3%)	12 (66.7%)	13 (35.1%)
**Years of professional practice**			
Mean (SD)	22.4 (7.9)	8.8 (8.1)	15.8 (10.5)
Median (IQR)	26 (8.0)	4 (15)	17 (22)
Range	1– 30	1–28	1–30
**Clinical experience in PAC (years)***			
Mean (SD)	2.7 (1.6)	8.4 (6.9)	5.5 (5.5)
Median (IQR)	3 (3.0)	4 1(4.25)	3 (2.5)
Range	0–5	0–25	0–25

Data are n (%), mean (SD), or median (IQR). *Before study start.

For peer review only - http://bmjopen.bmj.com/site/about/guidelines.xhtml

Table 2: Sociodemographic characteristics and reproductive history of participants* by provider

	Midwife	Physician	Total
**Age (years)**			
n	422	416	838
Mean (SD)	25.2 (5.9)	24.8 (5.4)	25.0 (5.6)
Range	14–45	12–41	12–45
14–19	60 (14%)	56 (14%)	116 (14%)
20–24	171 (40%)	167 (40%)	338 (40%)
25–29	101 (24%)	113 (27%)	214 (26%)
30–34	53 (13%)	51 (12%)	104 (12%)
35–39	28 (7%)	26 (6%)	54 (7%)
40–47	9 (2%)	3 (1%)	12 (1%)
**Marital status**			
n	433	426	859
Married or cohabiting	289 (67%)	292 (68%)	581 (68%)
Single	128 (30%)	124 (29%)	252 (29%)
Divorced/separated	9 (2%)	7 (2%)	16 (2%)
Widowed	7 (2%)	3 (1%)	10 (1%)
**Religion**			
n	433	426	859
Christian	427 (99%)	417 (98%)	844 (98%)
Muslim	6 (1%)	9 (2%)	15 (2%)
**Education**			
n	433	425	858
None	6 (1%)	2 (1%)	8 (1%)
Primary 1–4	11 (2%)	9 (2%)	20 (2%)
Primary 1–5	108 (25%)	121 (28%)	229 (27%)
Secondary	206 (48%)	186 (44%)	392 (46%)
Tertiary	102 (24%)	107 (25%)	209 (24%)
**Occupation**			
n	431	423	854
Unemployed	210 (49%)	197 (47%)	407 (48%)
Formal employment	73 (17%)	75 (18%)	148 (17%)
Self-employed	148 (34%)	151 (36%)	299 (35%)
**Gestational age based on clinical examination (weeks)**			
n	433	426	859
Mean (SD)	9.4 (2.2)	9.7 (2.1)	9.6 (2.2)
Range	1–12	3–12	1–12
**Number of previous pregnancies**			
n	433	425	858
Mean (SD)	1.75 (1.8)	1.8 (1.6)	1.8 (1.7)
Range	0–12	0–8	0–12
0	135 (31%)	101 (24%)	236 (27%)
1	94 (22%)	110 (26%)	204 (24%)
2–3	136 (31%)	157 (37%)	293 (34%)
4–5	58 (13%)	50 (11%)	108 (13%)
6–8	7 (2%)	7 (2%)	14 (2%)
9–12	3 (1%)	0	3
**Parity (live birth)**			
n	431	425	856
Mean (SD)	1.1 (1.4)	1.1 (1.3)	1.1 (1.3)
Range	0-10	0-7	0-10
0	205 (48%)	178 (42%)	383 (45%)
1	105 (24%)	111 (26%)	216 (25%)
2-3	94 (22%)	115 (27%)	209 (24%)

For peer review

4-5	23 (5%)	18 (4%)	41 (5%)
6-8	3 (1%)	3 (1%)	6 (1%)
9-11	1	0	1

Data are n (%) unless otherwise stated.
*Includes women lost to follow-up.
Internal drop-out range between 0–11 (0–2.6%), among the midwife and physician group, respectively.

Table 3: Outcome of treatment of incomplete abortion by provider

	Midwife	Physician	Risk difference (95% CI)	Adjusted difference (95% CI)*
Randomised and received intervention	446	444		
Per-protocol	409	401		
Complete abortion	390 (95.4%)	378 (94.3%)	1.0% (–4.1 to 2.0)	1.0% (–4.1 to 2.2)

*Adjusted for facility, age (<25 vs ≥25 years), marital status (single [single, widowed, divorced, separated] vs married or cohabiting), education (none or primary 1–8 vs secondary or tertiary), number of previous pregnancies (0 vs >1), and parity (para 0 vs ≥1).

Table 4: Reported symptoms following the treatment, by provider.

	**Midwife**	**Physician**	**Total**
**Bleeding since treatment**			
**N**	408	401	806
Less than normal menstrual bleeding	274 (67%)	268 (67%)	542 (67%)
Same as normal menstrual bleeding	78 (19%)	70 (18%)	148 (19%)
Havier than normal menstrual bleeding	56 (14%)	60 (15%)	116 (14%)
**Days bleeding**			
N	396	389	785
Mean	4.2 (2.0)	4.08 (1.7)	4.1 (2.0)
Range	1–14	1–10	1–14
**Pain following treatment**			
N	407	399	806
None	86 (21%)	84 (20%)	170 (21%)
Mild	200 (49%)	194 (49%)	394 (49%)
Moderate	81 (20%)	76 (19%)	157 (20%)
Severe	40 (10%)	45 (11%)	85 (10%)
**Unscheduled visit**			
N	403	397	800
Yes	20 (5%)	26 (6%)	46 (6%)
Vaginal bleeding or abdominal pain, or both	11 (3%)	18 (4%)	29 (4%)
Chill/fever or dizziness, or both	6 (1%)	6 (1%)	12 (1%)

Data are n (%) unless otherwise stated.
Internal drop-out range between 0–11 (0–2.6%), among the midwife and physician group, respectively.

For peer review only - http://bmjopen.bmj.com/site/about/guidelines.xhtml

Table 5: Reported secondary outcomes, by provider type.

	Midwife	Physician	Total
**How did you perceive the treatment**			
N	407	398	801
As expected	121 (30%)	130 (32%)	251 (31%)
Worse than expected	29 (7%)	27 (7%)	56 (7%)
Easier than expected	257 (63%)	241 (61%)	498 (62%)
**Felt safe (Acceptance of the method)**			
N	400	399	799
Yes	383 (96%)	378 (95%)	779 (97%)
No	17 (4%)	21 (4%)	38 (3%)
**Will recommend the treatment to a friend**			
N	400	399	799
Yes	383 (96%)	378 (95%)	761 (95%)
No	17 (4%)	21 (5%)	38 (5%)
**Received contraceptive counselling**			
N	405	401	806
Yes	395 (98%)	394 (98%)	789 (98%)
No	10 (2%)	7 (2%)	17 (2%)
**Accepted a contraceptive method**			
N	407	401	803
No	104 (26%)	91 (23%)	195 (24%)
Yes	303 (74%)	310 (77%)	613 (76%)
**What method did you chose**			
Injectable	118 (39%)	118 (38%)	236 (39%)
Pills	81 (27%)	85 (28%)	166 (27%)
Condoms	75 (25%)	76 (25%)	151 (25%)
Hormonal implants	24 (8%)	21 (7%)	45 (8%)
IUDs	3 (1%)	5 (2%)	8 (1%)
Permanent contraceptives	-	1	1

Data are n (%) unless otherwise stated.
Internal drop-out range between 0–11 (0–2.6%), among the midwife and physician group, respectively.

For peer review only

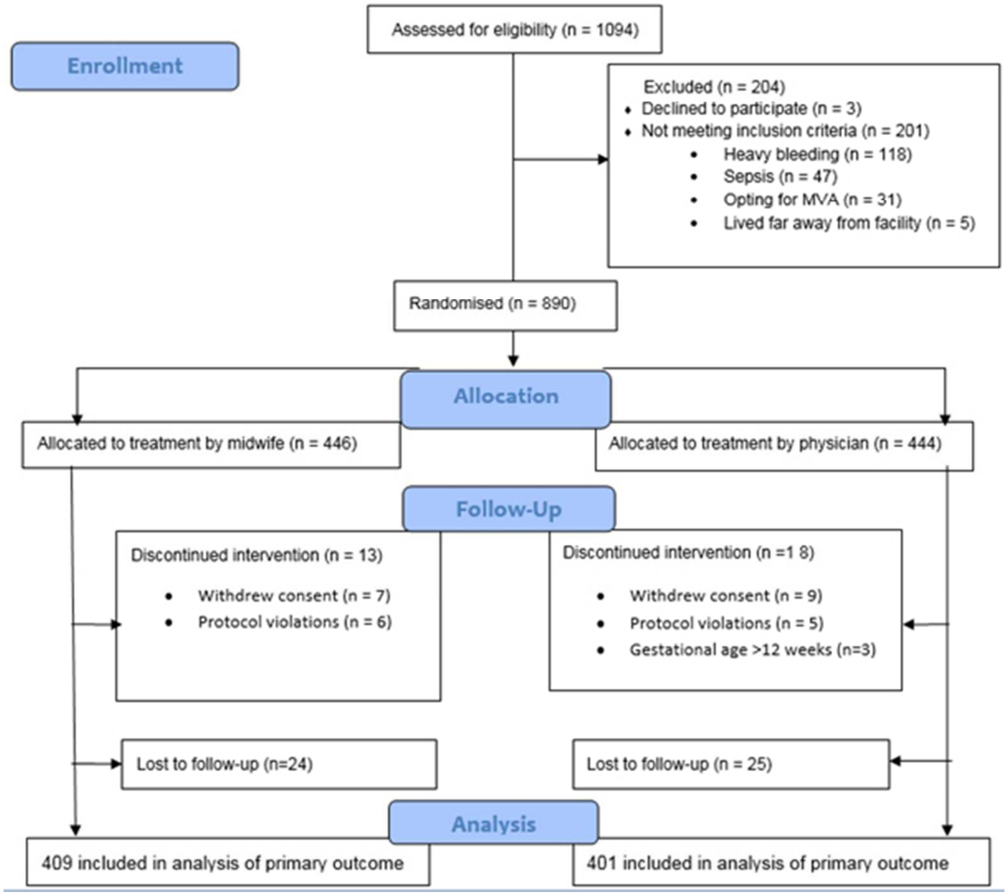

51x45mm (300 x 300 DPI)

only

Appendix 1. Symptoms followed the treatment of incomplete abortion, self-reported by women.

	Midwife	Physician	Total
**Self-reported side effects***			
**N**	409	401	810
None	201 (49%)	206 (51%)	407 (50%)
Abdominal pain	166 (41%)	139 (35%)	305 (38%)
Chills	51 (12%)	46 (12%)	97 (12%)
Nausea	32 (8%)	32 (8%)	64 (8%
Diarrhoea	27 (7%)	22 (6%)	49 (6%)
Vomiting	12 (3%)	17 (4%)	29 (4%)
Foil smelling vaginal or cervical discharge	9 (2%)	10 (2%)	19 (2%)

Data are n (%). *Several options was allowed to obtain.

For peer review only

For peer review only - http://bmjopen.bmj.com/site/about/guidelines.xhtml

CONSORT 2010 checklist of information to include when reporting a randomised trial*

Section/Topic	Item No	Checklist item	Reported on page No
**Title and abstract**			
	1a	Identification as a randomised trial in the title	TIDieR
	1b	Structured summary of trial design, methods, results, and conclusions (for specific guidance see CONSORT for abstracts)	TIDieR
**Introduction**			
Background and objectives	2a	Scientific background and explanation of rationale	TIDieR
	2b	Specific objectives or hypotheses	192-95, p5
**Methods**			
Trial design	3a	Description of trial design (such as parallel, factorial) including allocation ratio	197-98, p8
	3b	Important changes to methods after trial commencement (such as eligibility criteria), with reasons	TIDieR
Participants	4a	Eligibility criteria for participants	237-44, p7
	4b	Settings and locations where the data were collected	TIDieR
Interventions	5	The interventions for each group with sufficient details to allow replication, including how and when they were actually administered	TIDieR
Outcomes	6a	Completely defined pre-specified primary and secondary outcome measures, including how and when they were assessed	300-22, p9
	6b	Any changes to trial outcomes after the trial commenced, with reasons	344, p10
Sample size	7a	How sample size was determined	325, p9
	7b	When applicable, explanation of any interim analyses and stopping guidelines	n/a
Randomisation:			
Sequence generation	8a	Method used to generate the random allocation sequence	248, p7
	8b	Type of randomisation; details of any restriction (such as blocking and block size)	248, p7
Allocation concealment mechanism	9	Mechanism used to implement the random allocation sequence (such as sequentially numbered containers), describing any steps taken to conceal the sequence until interventions were assigned	249-53, p7
Implementation	10	Who generated the random allocation sequence, who enrolled participants, and who assigned participants to interventions	251-52 p7, 271-77, p8

For peer review only - http://bmjopen.bmj.com/site/about/guidelines.xhtml

Blinding	11a	If done, who was blinded after assignment to interventions (for example, participants, care providers, those assessing outcomes) and how	258-59, p7
	11b	If relevant, description of the similarity of interventions	
Statistical methods	12a	Statistical methods used to compare groups for primary and secondary outcomes	329-33, p9
	12b	Methods for additional analyses, such as subgroup analyses and adjusted analyses	329-33, p9
**Results**			
Participant flow (a diagram is strongly recommended)	13a	For each group, the numbers of participants who were randomly assigned, received intended treatment, and were analysed for the primary outcome	Figure 1
	13b	For each group, losses and exclusions after randomisation, together with reasons	Figure 1
Recruitment	14a	Dates defining the periods of recruitment and follow-up	288, p8, 359, p10
	14b	Why the trial ended or was stopped	na
Baseline data	15	A table showing baseline demographic and clinical characteristics for each group	Table 1
Numbers analysed	16	For each group, number of participants (denominator) included in each analysis and whether the analysis was by original assigned groups	Fig1, Table 1-5
Outcomes and estimation	17a	For each primary and secondary outcome, results for each group, and the estimated effect size and its precision (such as 95% confidence interval)	Table 3
	17b	For binary outcomes, presentation of both absolute and relative effect sizes is recommended	
Ancillary analyses	18	Results of any other analyses performed, including subgroup analyses and adjusted analyses, distinguishing pre-specified from exploratory	Table 3+line 374-77, 391- 94, p11
Harms	19	All important harms or unintended effects in each group (for specific guidance see CONSORT for harms)	Appendix1
**Discussion**			
Limitations	20	Trial limitations, addressing sources of potential bias, imprecision, and, if relevant, multiplicity of analyses	410-21, p12
Generalisability	21	Generalisability (external validity, applicability) of the trial findings	408-09, p12
Interpretation	22	Interpretation consistent with results, balancing benefits and harms, and considering other relevant evidence	433-37, p13
**Other information**			
Registration	23	Registration number and name of trial registry	350, p10
Protocol	24	Where the full trial protocol can be accessed, if available	Attached
Funding	25	Sources of funding and other support (such as supply of drugs), role of funders	512-15, p15

For peer review only

*We strongly recommend reading this statement in conjunction with the CONSORT 2010 Explanation and Elaboration for important clarifications on all the items. If relevant, we also recommend reading CONSORT extensions for cluster randomised trials, non-inferiority and equivalence trials, non-pharmacological treatments, herbal interventions, and pragmatic trials. Additional extensions are forthcoming: for those and for up to date references relevant to this checklist, see www.consort-statement.org.

For peer review only

For peer review only - http://bmjopen.bmj.com/site/about/guidelines.xhtml

TIDieR Template for Intervention Description and Replication

The TIDieR (Template for Intervention Description and Replication) Checklist*:

Information to include when describing an intervention and the location of the information

Item number	Item	Where located ** Primary paper (page or appendix number)	Other † (details)
	**BRIEF NAME**		
**1.**	Provide the name or a phrase that describes the intervention.	Page 1, Title	______________
	**WHY**		
**2.**	Describe any rationale, theory, or goal of the elements essential to the intervention.	Line 172-181, p 4-5	____________
	**WHAT**		
**3.**	Materials: Describe any physical or informational materials used in the intervention, including those provided to participants or used in intervention delivery or in training of intervention providers. Provide information on where the materials can be accessed (e.g. online appendix, URL).	Line 209-13, p6, line 261-270, p7	____________
**4.**	Procedures: Describe each of the procedures, activities, and/or processes used in the intervention, including any enabling or support activities.	Line 275-288, p8.	____________
	**WHO PROVIDED**		
**5.**	For each category of intervention provider (e.g. psychologist, nursing assistant), describe their expertise, background and any specific training given.	Line 265-70, p7 +Table 1	____________
	**HOW**		
**6.**	Describe the modes of delivery (e.g. face-to-face or by some other mechanism, such as internet or telephone) of the intervention and whether it was provided individually or in a group.	Line 279, p8	____________
	**WHERE**		

For peer review only - http://bmjopen.bmj.com/site/about/guidelines.xhtml

**7.**	Describe the type(s) of location(s) where the intervention occurred, including any necessary infrastructure or relevant features.	Line 199-209, p5-6, 261-65, p7, 275-78 p8	__________
	**WHEN and HOW MUCH**		
**8.**	Describe the number of times the intervention was delivered and over what period of time including the number of sessions, their schedule, and their duration, intensity or dose.	Line 283-288, p8	__________
	**TAILORING**		
**9.**	If the intervention was planned to be personalised, titrated or adapted, then describe what, why, when, and how.	N/A	__________
	**MODIFICATIONS**		
**10.**‡	If the intervention was modified during the course of the study, describe the changes (what, why, when, and how).	Line 216 p6, 343-44, p10	__________
	**HOW WELL**		
**11.**	Planned: If intervention adherence or fidelity was assessed, describe how and by whom, and if any strategies were used to maintain or improve fidelity, describe them.	Line 255-58, 292-99 p8	__________
**12.**‡	Actual: If intervention adherence or fidelity was assessed, describe the extent to which the intervention was delivered as planned.	Table 3 and 4	__________

** **Authors** - use N/A if an item is not applicable for the intervention being described. **Reviewers** – use '?' if information about the element is not reported/not sufficiently reported.

† If the information is not provided in the primary paper, give details of where this information is available. This may include locations such as a published protocol or other published papers (provide citation details) or a website (provide the URL).

‡ If completing the TIDieR checklist for a protocol, these items are not relevant to the protocol and cannot be described until the study is complete.

* We strongly recommend using this checklist in conjunction with the TIDieR guide (see *BMJ* 2014;348:g1687) which contains an explanation and elaboration for each item.

* The focus of TIDieR is on reporting details of the intervention elements (and where relevant, comparison elements) of a study. Other elements and methodological features of studies are covered by other reporting statements and checklists and have not been duplicated as part of the TIDieR checklist. When a **randomised trial** is being reported, the TIDieR checklist should be used in conjunction with the CONSORT statement (see www.consort-statement.org) as an extension of **Item 5 of the CONSORT 2010 Statement.** When a **clinical trial protocol** is being reported, the TIDieR checklist should be used in conjunction with the SPIRIT statement as an extension of **Item 11 of the SPIRIT 2013**

TIDieR checklist

For peer review only

Statement (see www.spirit-statement.org). For alternate study designs, TIDieR can be used in conjunction with the appropriate checklist for that study design (see www.equator-network.org).

For peer review only

For peer review only - http://bmjopen.bmj.com/site/about/guidelines.xhtml

BMJ Open

Post-abortion care with misoprostol - equally effective, safe and accepted when administered by midwives compared to physicians: a randomised controlled equivalence trial in a low-resource setting in Kenya

Journal:	*BMJ Open*
Manuscript ID	bmjopen-2017-016157.R2
Article Type:	Research
Date Submitted by the Author:	08-Aug-2017
Complete List of Authors:	Makenzius, Marlene; Karolinska Institutet Department of Public Health Sciences, Global Health (IHCAR)<br>Oguttu, Monica; Kenya Medical Education Trust - KMET<br>klingberg-allvin, marie; Hogskolan Dalarna Akademin Utbildning Halsa och Samhalle<br>Gemzell, Kristina; Karolinska Universitetssjukhuset, Dept. of Women´s and Children´s Health<br>Odero, Theresa; College of Health Sciences, School of Nursing Sciences, University of Nairobi<br>Faxelid, Elisabeth; Karolinska Institutet Department of Public Health Sciences
**Primary Subject Heading**:	Global health
Secondary Subject Heading:	Medical education and training, Obstetrics and gynaecology, Sexual health, Reproductive medicine, Health policy
Keywords:	EDUCATION & TRAINING (see Medical Education & Training), Community gynaecology < GYNAECOLOGY, Reproductive medicine < GYNAECOLOGY, Health policy < HEALTH SERVICES ADMINISTRATION & MANAGEMENT, Maternal medicine < OBSTETRICS

SCHOLARONE™
Manuscripts

TITLE

Post-abortion care with misoprostol - equally effective, safe and accepted when administered by midwives compared to physicians: a randomised controlled equivalence trial in a low-resource setting in Kenya.

Short title: Post-abortion care with misoprostol: A randomised controlled trial in a low-resource setting, Kenya

AUTHORS

***Marlene Makenzius (MM),** Postdoctor monitoring the RCT at site, PhD, MPH, CNM
Dept. of Public Health Sciences Global Health (IHCAR), Karolinska Institutet
Postal address: Tomtebodavägen 18A, SE-171 77 Stockholm, Sweden
Phone: +46 761131 076, +254 7342 47498
marlene.makenzius@folkhalsomyndigheten.se; marlene.makenzius@ki.se

Monica Oguttu (MO), PI, Director at KMET, PhD, RM, RN
Kenya Medical Education Trust - KMET
Postal address: Tom Mboya Estate, P.O. Box 6805 – 40103, Kisumu, Kenya
+254 701 839 115 or 721 845948

Marie Klingberg-Allvin (MKA), Professor/Pro Vice-Chancellor, PhD, RM, RN
School of Education, Health and Social studies, Dalarna University
Postal address: Dalarna University, SE-791 88 Falun, Sweden and Dept. of Women´s and Children´s Health, Karolinska Institutet

Kristina Gemzell-Danielsson (KMD), Professor, MD, PhD, Chair Div. of Obstetrics and Gynecology
Dept. of Women´s and Children´s Health, Karolinska Institutet
Postal address: WHO-centre, C1:05, Karolinska University Hospital, SE-171 76 Stockholm, Sweden
Phone: +46 8 517- 72128 (or -79539)

Theresa M A Odero (TO), PhD-stud, MSc, RM, RN
College of Health Sciences, School of Nursing Sciences, University of Nairobi
Postal address: P O Box 19676, code 00202 KNH, Nairobi, Kenya

Elisabeth Faxelid (EF), PI, Sweden), Prof., PhD, RM, RN
Dept. of Public Health Sciences Global Health (IHCAR), Karolinska Institutet
Postal address: Tomtebodavägen 18A, SE-171 77 Stockholm, Sweden

*Corresponding author

I Marlene Makenzius The Corresponding Author of this article contained within the original manuscript which includes any diagrams & photographs within and any related or stand alone film submitted (the Contribution") has the right to grant on behalf of all authors and does grant on behalf of all authors, a licence to the BMJ Publishing Group Ltd and its licencees, to permit this Contribution (if accepted) to be published in the BMJ and any other BMJ Group products and to exploit all subsidiary rights, as set out in our licence set out at: http://www.bmj.com/about-bmj/resources-authors/forms-policies-and-checklists/copyright-open-access-and-permission-reuse. Please tick one or more boxes as appropriate:

YES, I am one author signing on behalf of all co-owners of the Contribution.

For peer review only

Author's contribution

The lead author for this manuscript was Marlene Makenzius

Study design and planning: Kristina Gemzell Danielsson, Elisabeth Faxelid, Marie Klingberg Allvin and Monica Oguttu.
Training and implementation of the study: Marie Klingberg Allvin, Monica Oguttu, Kristina Gemzell Danielsson, Elisabeth Faxelid, Theresa Odero and Marlene Makenzius.
Data Collection: Marlene Makenzius, Monica Oguttu and Theresa Odero
Data analysis: Marlene Makenzius, cross-checked by a senior statistician, Ulla Romild, at the National Agency of Public Health in Sweden.
Data interpretation: Marlene Makenzius, Elisabeth Faxelid, Kristina Gemzell Danielsson, Marie Klingberg Allvin, Monica Oguttu and Theresa Odero.
Literature search: Marlene Makenzius
Writing: Marlene Makenzius
Editing and proof-reading: Kristina Gemzell Danielsson, Elisabeth Faxelid, Marie Klingberg Allvin, Monica Oguttu, Theresa Odero, and Scribendi (language editing).

ABSTRACT

Objective To assess the effectiveness of midwives administering misoprostol to women with incomplete abortion seeking post-abortion care (PAC), compared with physicians.

Design A multicentre randomised controlled equivalence trial. The study was not masked.

Settings Gynaecological departments in two hospitals in a low-resource setting, Kenya.

Population Women (n=1094) with incomplete abortion in the first trimester, seeking PAC during 1 June 2013 to 31 May 2016. Participants were randomly assigned to receive treatment from midwives or physicians. 409 and 401 women in the midwife and physician groups, respectively, were included in the per-protocol analysis.

Interventions 600µg misoprostol orally, and contraceptive counselling by a physician or midwife.

Main outcome measures Complete abortion not needing surgical intervention within 7–10 days. The main outcome was analysed on the per-protocol population with a generalised estimating equation model. The predefined equivalence range was –4 % to 4%. Secondary outcomes were analysed descriptively.

Results The proportion of complete abortion was 94.8% (768/810), 390 (95.4%) in the midwife-group, and 378 (94.3%) in the physician-group. The proportion of Incomplete abortion was 5.2% (42/810), similarly distributed between midwives and physicians. The model-based risk difference for midwives versus physicians was 1.0% (–4.1 to 2.2). Most women felt safe (97%; 779/799), 93% (748/801) perceived the treatment as expected/easier than expected. After contraceptive counselling the uptake of a contraceptive method after 7-10 days occurred in 76% (613/810). No serious adverse events were recorded.

Conclusions Treatment of incomplete abortion with misoprostol provided by midwives is equally effective, safe, and accepted by women as when administered by physicians in a low-resource setting. Systematically provided contraceptive counselling in PAC is effective to mitigate unmet need for contraception.

The trial was registered at ClinicalTrials.gov, NCT01865136

For peer review only - http://bmjopen.bmj.com/site/about/guidelines.xhtml

Strengths

- Despite a restrictive abortion law the follow-up rate was considered high.
- A large sample size
- A rigorous project design and trial implementation was adopted in collaboration with Ministry of Health, different cadres of health care providers, leaders, NGOs, and universities.

Limitations

- District hospitals are usually more advanced regarding staffing and resources and might therefore not be representative of the small health dispensaries located in the most remote areas of the region and other parts of the country.
- Both midwives and physicians were working in the same health facility and therefore there could have been interaction between the two sets of providers in post-abortion care.
- The contraceptive up-take was high at follow-up (7-10 days), but lacks long-term follow-up.

For peer review only

INTRODUCTION

Although safe, simple and effective evidence-based interventions exist, about 22 million unsafe abortions take place each year worldwide.[1] An estimated 47,000 women die annually from complications resulting from the resort to unsafe practices for termination of pregnancy[1], one of the leading causes of death among young women in sub-Saharan Africa.[1-6] Unsafe abortion is defined by WHO as a procedure performed under conditions that fail to meet the minimal medical standards and/or by untrained persons.[3]

Access to post-abortion care (PAC) for treatment of the complications of unsafe abortion and spontaneous abortion (i.e. all fetal or placental tissue have not been expelled and severe bleeding, or infection occur and prompt medical attention is required), has increased over decades.[1] However women in many countries still do not have access to this life saving care or are mistreated when they seek it.[6] The costs for PAC constitute a significant financial burden on public healthcare systems.[1,7-9]

An estimated 465,000 induced abortions occurred in Kenya in 2012, 48 induced abortions in 1000 women of reproductive age (15–49 years), and in some regions 35% of maternal deaths are attributed to unsafe abortions.[2] Because of the restrictive abortion law in Kenya[10], limited access to quality health care, and stigma most of the Kenyan abortions were done outside authorized facilities and thus considered unsafe.[2] An estimated 120,000 women were treated for complications resulting from unsafe abortion in 2012.[2] Moreover, the unmet need for family planning remains high in Kenya[2,11,12] since 71% of women seeking PAC did not use a contraceptive method prior to becoming pregnant.[2,11] Contraception has clear health benefits as prevention of unintended pregnancies results in a subsequent decrease in maternal and infant mortality and morbidity.[11,13]

Even though there are no restrictions to treat abortion complications, most providers in Kenya have not been trained in PAC, in accordance with the WHOs technical and policy guidance.[3]

For peer review only - http://bmjopen.bmj.com/site/about/guidelines.xhtml

PAC is an integral component of comprehensive abortion care (CAC), and is often used in settings where abortion is legally restricted.[13,14] Essential elements are community and service provider partnership, counselling, treatment of incomplete and unsafe abortion, contraceptive services, and linkages to reproductive and other health services.[13] The prostaglandin E_1 analogue misoprostol is effective treatment for incomplete abortion in the first trimester, in PAC[3, 13-16], and no significant differences has been reported in the effectiveness between misoprostol and manual vacuum aspiration (MVA) for the treatment of incomplete abortion.[14,15] Misoprostol simplifies the procedure, compared to MVA, which is more intrusive.[13-16] Even though the use of MVA and misoprostol is increasing in Kenya, other less safe procedures remain common, reinforced by the mismatch between where maternal mortality occurs and where health workers are.[17] In addition, regulations restrict misoprostol prescription and supervision to physicians, while nurses and midwives tend to have a subordinate role compared to physicians.[1,17]

Moving beyond specialist doctors to involve a wider range of health workers is an increasingly important public health strategy to reduce maternal mortality in low resource settings.[18-22] Trained nurses and midwives have in some RCTs been shown to be able to provide safe medical abortion[23,,24] and to treat incomplete abortion as effectively as physicians.[25] It is estimated that the global deficit of skilled health-care professionals will reach 12.9 million by 2035.[26] Such shortages are especially critical in regions of the world that also have a high burden of unsafe abortion and related mortality. Thus expanding health worker roles, opens a window for improving access to safe PAC.[20,26] The range of safe and effective options, including non-surgical methods, can facilitate evidence-based decision-making and adaptation to the local context, resources and public health needs.[1,26-29]. The aim with this trial was to assess the effectiveness of midwives diagnosing incomplete abortion and administering misoprostol to women needing PAC, compared with physicians.

For peer review only - http://bmjopen.bmj.com/site/about/guidelines.xhtml

METHODS

The study was a multicentre randomised parallel-group trial, reported in accordance with the CONsolidated Standards of Reporting Trials (CONSORT) guidelines for non-inferiority and equivalence randomised trials.[30] The project was conducted at the Jaramogi Oginga Odinga Teaching and Referral Hospital (JOOTRH) and Kisumu County Hospital (KCH) in Kisumu County, western Kenya. Regional variations in abortion incidence exist in Kenya, and the rate in this region (Nyanza/Western) was among the highest, 63 induced abortions per 1000 women of reproductive age (15-49 years), compared with 48 for the country.[2,17] Consequently, severe complications from unsafe abortion were common, and associated with delays in seeking health care being managed in a lower level facility, and being referred to an upper level facility.[17] The superior regime to treat first trimester incomplete abortion was MVA (62%), similar to the country (65%), and medical abortion catered for only 11% .[17] In the region about 37% of women (15-49) had ever used a contraceptive method compared to 46% in the country.[17]

Kisumu is the third largest city in Kenya and has a population of about 500,000 people. The project was implemented in the gynaecological ward within the Department of Obstetrics and Gynaecology at the two facilities (June 1th, 2013 until May 31, 2016). Altogether the two facilities admit between 20-32 women with incomplete abortions per month.

The study was designed and coordinated by researchers at the Department of Public Health Sciences, Global Health (IHCAR), and Women's and Children's Health, Karolinska Institutet, Stockholm, Sweden; Kisumu Medical Education Trust (KMET); University of Nairobi; and Moi University, Eldoret, Kenya; and at JOOTRH and KCH in Kisumu, Kenya.

Research question and outcome measures was constructed by the researcher team, and health care providers at the study facilities who were experienced in PAC, in order to strengthen the validity. The preliminary results was continuously reported during the study period to the two facilities by the study coordinator to encourage the provider's work, who also used the information to encourage the participating women. The outcome and acceptability of the intervention was evaluated by the research assistants at follow-up, and the women were advised to seek care ahead of the appointment if they experienced unexpected symptoms/burden.

We obtained ethical approval from the JOOTRH Ethical Review Committee (diary number ERC42/13), and the Swedish regional Ethics Committee in Stockholm (2013/902-31/1). All the patients gave written informed consent. The participants could withdraw from participation at any time without consequences. Consent forms were translated into the local languages Kiswahili and Luo and back

For peer review only - http://bmjopen.bmj.com/site/about/guidelines.xhtml

translated into English to ensure accurate translation. The form was read to each participant and those unable to write marked the form with a thumb print, and a witness signed to indicate that the study had been explained to the participant and that she understood and accepted to participate.

Inclusion and exclusion criteria

Inclusion criteria were women with signs of incomplete abortion, based on clinical assessments (i.e., positive pregnancy test, bleeding, contractions and lower abdominal pain, open cervical os, and partial expulsion) and physical examination (including vaginal examination), but without the use of ultrasound. Exclusion criteria were complete abortion (no bleeding or contractions), a uterine size estimated over 12 weeks of gestation, suspected ectopic pregnancy (severe abdominal pain), unstable haemodynamic status, signs of pelvic infection or sepsis, and known allergy to misoprostol.

Randomisation and masking

Eligible women who consented to participate were randomly allocated to a midwife (intervention) or standard care with a physician (control) for diagnosis and treatment. The randomisation was done in blocks of eight and was stratified for study site. We used a computer random number generator to generate a list of codes from 1 to 890 and each code was linked to one of the two study groups. Sequentially numbered, opaque, sealed envelopes, each containing a random allocation, were prepared at the coordinating department of KMET, and later opened in consecutive order by the research assistants after obtaining written consent. Data management was organised locally at the coordinating department at KMET, Kisumu.

The study coordinator checked case report forms for accuracy, and corrected them if needed after discussion with research assistants, and did continuous process evaluation. The providers received support and guidance throughout the study period by the study coordinator. The study was not masked to the study participants or providers.

Procedures

Eligible providers for participation were midwives (n = 19) and physicians (n = 18), Table 1, involved in PAC at the two facilities. The providers were trained by the research team in a 5-day training program according to Kenyan and the WHO standardised PAC training module.[3,31,32] The training was facility-based and both theoretical and practical, and repeated two times during the project period in order to cover all/new staff. This training included knowledge on diagnosing incomplete abortions,

For peer review only - http://bmjopen.bmj.com/site/about/guidelines.xhtml

treatment with misoprostol and MVA, and post-abortion contraceptive counselling as well as interview technique. The healthcare facilities were equipped by the project to provide basic and emergency obstetric services and were estimated to have sufficient staff and resources required for the trial. The procedure and the instruments were pilot tested at the two hospitals before the study started. The first two months was set to a run-in phase, in which the facilities were given time to set the routine among staff for screening, enrolment, and follow-up, as well as to emancipate space to store a large number of protocol-records safely, and to provide counselling in a secluded room. Minor revisions were made to the instruments and the recruitment procedure.

Out of the 19 midwives, four per facility were trained as research assistants in the study. They were responsible to oversee and monitor supply in stock (misoprostol and contraceptives such as condoms, pills, Depo-Provera [medroxyprogesterone acetate injectable], hormonal implants and intra uterine devices [IUD]). In addition, the research assistants were responsible for the follow-up visit including assessment of treatment outcome and contraceptive uptake, and performing MVA when necessary.

Women admitted with signs of incomplete abortion were first screened by a research assistant, based on self-reported last menstrual period, pregnancy test and symptoms. Eligible women who consented to participate were randomly allocated to the midwife or physician group. The clinical assessment included (Table 2); the last menstrual period, obstetric and gynaecological history, pelvic examination including any signs of genital infections, cervical status, bleeding, and size of the uterus. Ultrasound was not used.

Each participant was given one single dose of 600 µg misoprostol orally. Additionally, they were offered analgesics (ibuprofen or paracetamol) and oral antibiotics given according to local guidelines for PAC. For monitoring purposes, participants were advised to stay at the clinic for 4 to 12 hours after swallowing the misoprostol tablets. Before discharge, all women were offered contraceptive counselling and provided with a contraceptive method if they wanted, and given a follow-up appointment after 7–10 days.

Participants received detailed information about bleeding, pain expected, abnormal symptoms after treatment (severe pain, fever, and foul smelling vaginal discharge), and were informed about the importance of seeking care or consulting on the toll-free line open 24/7 if such symptoms occurred. Side-effects were recorded (Appendix 1). The women were offered reimbursement for travel costs as an encouragement to come for follow-up. All women received an SMS reminder two days before the scheduled follow-up appointment. Women who did not show up were contacted by the research

For peer review only

assistant using a cell phone provided by the project, and the woman was given a new appointment and encouraged to attend.

Outcomes

The pre-specified primary outcome was complete abortion, requiring no further medical or surgical intervention, Table 3. A symptom diary card was used to assess secondary outcomes following the treatment (Table 4)

(i) daily bleeding in relation to normal menstrual bleeding,
(ii) pain following the treatment,
(iii) unscheduled visits at a healthcare facility and the reason,
(iv) women's acceptability of the treatment was recorded by three questions "How did you perceive the treatment procedure", "Did you feel safe after the treatment", and will you recommend the treatment to a friend or a relative"

Contraceptive uptake (part of comprehensive PAC) was an additional outcome recorded by three questions "Did you receive contraceptive counselling, "Did you accept a contraceptive method", "What method did you chose" (Table 5).

Statistical analyses

The sample size was calculated with the objective of showing two-sided equivalence assuming that the rate of incomplete abortions could be 4% and would apply to both types of providers.[26,29] A pre-defined acceptable difference in completion rate between the two providers ranged from –4% to +4%. The margin was based on previous studies and clinically and statistically important differences as well as ethical criteria, cost, and feasibility.[24,26,30] To establish equivalence with a power of 80% and two-sided 95% CI, the required sample size was 400 per group. Compensation for about 10% loss to follow-up gave a total sample size of 888. Background characteristics were analysed and presented with descriptive statistics, as per the intention to treat, i.e. patients correctly allocated to study group and having received treatment. Main- and secondary outcomes were analysed as per-protocol, since they were measured at follow-up.

Analysis of the primary outcome (complete abortion) was made with a generalised estimating equation model to estimate the risk difference between the two providers with (two-sided) 95% CI. If the CI of the risk difference between the two groups fell within the predetermined margin of equivalence (-4% to +4%), the two types of providers were considered equivalent. P values of 0.05 or less were considered statistically significant. Additionally, we adjusted for the following covariates: facility, age (<25 vs ≥25 years), marital status (single vs married or cohabiting), education (none, primary 1–4, or 5–8 vs secondary or tertiary), number of previous pregnancies (1 vs >1), and live births (0 vs ≥1). We estimated the adjusted risk difference as the predicted risk difference at the

For peer review only - http://bmjopen.bmj.com/site/about/guidelines.xhtml

average of all included covariates. Safety parameters of the procedure were viewed descriptively without any formal statistical testing. The study protocol was not changed after the run-in period. All data were entered continuously throughout data collection and cleaned in IBM SPSS Statistics for windows version 22.0, and we did all the statistical analysis within this software. The trial is registered at ClinicalTrials.gov, number NCT01865136.

Role of the funding source

The authors designed this an investigator-initiated trial, in collaboration with healthcare providers and management at the study setting. The funders did not play any role in the study design or in the collection, analysis, and interpretation of data. All authors had full access to the data, and the first author regularly reported preliminary results to the whole research team. The corresponding author had final responsibility for the decision to submit the manuscript for publication.

RESULTS

From 1 June 2013 to 31 May 2016, we assessed 1094 women with signs of incomplete abortion for eligibility for participation in this study (Figure 1). Finally, a total of 810 women, 409 in the midwife group and 401 in the physician group received misoprostol treatment with misoprostol and were followed-up. All those were included in the per-protocol analysis of the main outcome.

Figure: Trial profile

Among the providers, the midwives had longer work experience than the physicians; on the other hand, the physicians were more experienced in PAC than the midwives in PAC (Table 1).

The baseline characteristics such as sociodemographic background and reproductive history were similarly distributed between the two groups (Table 1). The mean duration of gestational age based on clinical examination was 9.6 weeks (range 1–12 weeks; Table 2).

Sub-analyses showed slightly higher proportions of young (< 25 years), single, unemployed, and nulliparous women among those who dropped out of the study compared to those who came for the follow-up visits. There were no differences in gestational age or education levels between the two groups. None of the results of the sub-analyses were statistically significant (p > 0.05).

Main outcome

The overall proportion of complete abortion was 94.8% (768/810). As shown in Table 3, 390/409 (95.4%) women in the midwife group and 378/401 (94.3%) women in the physician group had complete abortions (Table 3). The model-based risk difference for midwife versus physician group

For peer review only - http://bmjopen.bmj.com/site/about/guidelines.xhtml

was 1.0% (–4.1 to 2.0). There were 42/810 incomplete abortions (5.2%), 19/409 (4.6%) in the midwife group and 23/401 (5.7%) in the physician group. Among the 42 women with incomplete abortions, 25 were treated with MVA and 17 were treated with a repeat dosage of misoprostol after they completed the follow-up assessment. Among the 25 women who obtained MVA, 2 were treated with a repeat dosage of misoprostol prior to MVA. No serious adverse events were recorded.

Secondary outcomes

Table 4 shows that most women (690; 86%) reported bleeding less than or the same as normal menstrual bleeding after treatment, and the mean number of bleeding days was 4.1 (SD 2.0; range, 1–14). A majority (564/806; 70%) of the women reported no or mild pain after treatment, and 6% (46/806) women reported unscheduled visits. Vaginal bleeding and abdominal pain were reported as reasons for the unscheduled visits. Most women perceived the treatment to be as expected or easier than expected (748/801; 93%), and 97% felt safe during the procedure (779/799). Additionally, 95% (761/799) women stated that they would recommend the treatment to a friend. There were no significant ($p < 0.05$) differences in the secondary outcomes between the groups. 407/810 women reported no side-effects following the treatment, while 402/810 women reported symptoms; abdominal pain, chills, nausea, diarrhoea, vomiting, foul smelling vaginal and/or cervical discharge, and were similar in both groups (Appendix 1).

Contraceptive up-take

Contraceptive counselling was provided to 789 of the 810 (98%) women; of these, 613 (76%) accepted a contraceptive method of their own choice (Table 5). There were no significant differences in the contraceptive up-take between the study groups ($p = .342$). The following choices of contraceptive methods were offered, and similarly distributed between the two types of providers: injectable (236/613; 39%), pills (166/613; 27%), condoms (151/613; 25%), hormonal implant (45/613; 8%), IUD (8/613; 1%), and permanent contraception (1/613; 0.16%).

DISCUSSION

The high proportion of complications related to unsafe abortion continues to pose a major public health challenge in Kenya.[17] Our finding shows that treating women with incomplete abortion (≤12 weeks of gestation), with misoprostol was equally effective and safe when provided by midwives or physicians in a low-resource setting in Kenya. In addition, the treatment is highly accepted among the treated women, and three out of four women chose to start a contraceptive method, when contraceptive counselling is systematically provided in post-abortion care (PAC).

For peer review only - http://bmjopen.bmj.com/site/about/guidelines.xhtml

For peer review only

The overall complete abortion rate was 94.8%, which is comparable with the results from other studies that have used the same regimen (94.5–96.7%)[16,21,24,25] The result emphasizes that the safety of using misoprostol for treatment of incomplete abortion in low-resource settings, can be performed by trained midwives without the routine use of ultrasonography before or after abortion.

Of the women with incomplete abortion (42/810), 25 were treated with MVA and 17, with repeated dosage of misoprostol, similarly distributed between the two provider types. Repeat dosage of misoprostol is apparently an alternative for MVA when the first dosage of misoprostol has failed. This could be compared with results from a similar study from Uganda where all women with incomplete abortion were treated with MVA (36/955).[25]

A notable additional finding was the frequency of women with sepsis, which was a criterion for ineligibility in this study (47/1094). This proportion is higher, that has been seen in other African countries[2,33] This might be an important reflection of the magnitude of unsafe abortion and the immense poor state of reproductive health access for women in Kenya. There is no doubt that there is room for improving timely and accessible CAC services, in particular PAC, in Kenya.

Although these results clearly show that misoprostol is a simple, efficient and desirable method, which is highly accepted by women, implementation of PAC in Kenya is hampered by the lack of national clinical guidelines.[2,33] Laws and policies governing access to safe abortion in Africa vary, ranging from very restrictive, such as in Kenya, where abortion is permitted only to save the life of the mother, to liberal, like in South Africa, where abortion is permitted through the 12 week of pregnancy or later.[1] Stigmatizing attitudes and beliefs surrounding abortions in the society, not least among healthcare providers[20,29], contribute to women's delay in seeking timely care for abortion-related complications.[2]

Kenya has a policy framework to prevent maternal mortality and morbidity. However, this policy framework excludes unsafe abortion, which is addressed through the provision of PAC.[2,17] In 2012, the Ministry of Health (MH) developed clinical guidelines on prevention of morbidity and mortality from unsafe abortions in Kenya, but the document was arbitrarily withdrawn in 2013. A task force was commissioned by the Director of Medical Services to consult and revise the guidelines, and the work was finalised in September 2015, but since then, signing of the document has stalled. Simultaneously, women seeking treatment for complications arising from unsafe abortions is on the rise.[1,2]

For peer review only - http://bmjopen.bmj.com/site/about/guidelines.xhtml

New guidelines may not only contribute to increase the quality and effectiveness to treat complications of unsafe abortion, it may also encourage the prevention of unintended pregnancy. Unmet contraceptive need and contraceptive failure will consistently be associated with high rates of unplanned and unwanted pregnancies, forcing many women to resort to unsafe abortion with a high rate of complications including infertility, long term morbidity and death.[33] The current result shows that systematically provided contraceptive counselling to PAC-seeking women is feasible and effective, as three out of four women accepted a contraceptive method. However, the use of modern LARC methods, such as hormonal implants and IUDs, was extremely low, in comparison to injectables. This indicates a pronounced need for concerted efforts to urgently reach women who have an unmet contraceptive need by giving them access to post-abortion contraceptive counselling and modern methods. Mitigating the unmet need for contraception could be the key to reducing numbers of subsequent unintended pregnancies and unsafe abortions, as well as maternal morbidity and mortality.

The strengths of the study lie in its large sample size, high follow-up rate and the socio-demographic characteristics of the women (i.e. heterogeneous, though overall low socioeconomics similar to those of the population in the country[17]). There are however weaknesses of the study to be considered. First, the midwives had more years of professional experience than the physicians, but the physicians were more experienced in PAC. No more information was given in detail about their working experience, however, all the providers within the two facilities received the same training in the treatment of incomplete abortion. Secondly, although the socioeconomic and demographic characteristics of study participants were representative of women in fertile ages in Kisumu County, they might not be representative of women living in the most remote rural areas of this county. Third, district hospitals are usually more advanced regarding staffing and resources and might therefore not be representative of the small health dispensaries located in remote areas of the region. Fourth, both types of providers worked in the same hospital and in the same environment. We cannot rule out interactions between the two provider groups, which might have led to a convergence of outcome. Fifth, drop-outs to follow-up was a challenge, as well as the lack of long-term follow up for the contraceptive up-take.

Though we encouraged women to come for follow-up by paying for the local transport, sent SMS reminders ahead of the scheduled appointment, and called the patient if they did not show up, 49 of the 859 women did not come for the follow-up. Some of the women did not have a phone and were thus not reachable, and some of the women who were contacted by phone did not find it necessary to attend the follow-up, as they considered themselves to have recovered without any side effects. In addition, the adverse event profile matches the known adverse profile of the drug and there were

For peer review only - http://bmjopen.bmj.com/site/about/guidelines.xhtml

no striking differences between the two categories of providers in this regard. Loss to follow-up could also be related to abortion stigma, which is present within this setting and other settings where abortion is restricted.[20] Women seeking PAC have previously reported shame and guilt, and they don't want to be associated with the abortion clinic and its activities once the abortion issue is completed.[20,29] Therefore, in coherence with Osur et al. (2013), instead of considering drop-outs criteria as bias, this might rather be an important reflection of the stigma surrounding abortion.

Future research needs to investigate if the results of this study are replicable in rural primary health care settings, such as health centres and dispensaries since it may differ from health care at district level. Moreover, future research needs to address long-term follow-up on contraceptive adherence after PAC. In spite of the convincing rationality in PAC of preventing future unplanned pregnancies by providing post-abortion contraceptive services, this component remains one of the weakest parts of post-abortion care.[1,2,13,33]

In conclusion, treatment of incomplete abortion with misoprostol when provided by midwives is equally effective, safe, and accepted as when administered by physicians in a low-resource setting. Systematically provided contraceptive counselling composed with a wide range of contraceptive choices in PAC is effective to mitigate unmet need for contraception. In areas with shortages of physicians and scarce resources, midwives would greatly increase women's access to safe and highly accepted PAC. These findings are timely and needed to accomplish standards and guidelines regarding CAC and PAC in Kenya and in other African countries lacking such strategies.

Data sharing

Additional data is available by emailing marlene.makenzius@ki.se

Declaration of interests

We declare no competing interests.

Funds

The Swedish Research Council (2012-06114; 2011-03369), and the Swedish Research Council on Health, Working Life and Welfare (2015-01398_3).

Acknowledgements

The authors acknowledge Caroline Nyandat and Sam Owoko for training and providing technical support, the data assistants Christine Apondi and Beatrice Otieno, the staff of Ministry of Health in Kisumu, Dr Paul Mitei, and Prof. Edwin Were, Moi University, who made this project possible and

For peer review only - http://bmjopen.bmj.com/site/about/guidelines.xhtml

For peer review only

facilitated its implementation. Thanks to Ulla Romild at the National Agency of Public Health, Sweden, for statistical advice. Finally, we thank all the women who participated in our study, and all the providers involved in the study who remained committed and supportive, throughout.

REFERENCES

1. Sedgh G, Bearak J, Singh S, et al. Abortion incidence between 1990 and 2014: global, regional, and subregional levels and trends. *Lancet* 2016; **388**(10041): 258–67.
2. Ziraba AK, Izugbara C, Levandowski BA, et al. Unsafe abortion in Kenya: a cross-sectional study of abortion complication severity and associated factors. *BMC Pregnancy Childbirth* 2015; **15**: 34.
3. Safe abortion: technical and policy guidance for health systems, second edition. Geneva: World Health Organization; 2012. http://apps.who.int/iris/bitstream/10665/70914/1/9789241548434_eng.pdf (accessed July 20 2017).
4. Singh S. Hospital admissions resulting from unsafe abortion: estimates from 13 developing countries. *Lancet* 2006; **368**(9550): 1887–92.
5. Mayi-Tsonga S, Oksana L, Ndombi I, Diallo T, de Sousa MH, Faundes A. Delay in the provision of adequate care to women who died from abortion-related complications in the principal maternity hospital of Gabon. *Reprod Health Matters* 2009; **17**(34): 65–70.
6. Temmerman Marleen, Khosla Rajat, Laski Laura, Mathews Zoe, Say Lale. Women's health priorities and interventions *BMJ* 2015; 351 :h4147
7. Johnston HB, Gallo MF, Benson J. Reducing the costs to health systems of unsafe abortion: a comparison of four strategies. *J Fam Plann Reprod Health Care* 2007; **33**(4): 250–7.
8. Vlassoff M, Walker D, Shearer J, Newlands D, Singh S. Estimates of health care system costs of unsafe abortion in Africa and Latin America. *Int Perspect Sex Reprod Health* 2009; **35**(3): 114–21.
9. Prada E, Maddow-Zimet I, Juarez F. The cost of postabortion care and legal abortion in Colombia. *Int Perspect Sex Reprod Health* 2013; **39**(3): 114–23.
10. National Council for Law Reporting. Constitution of Kenya 2010 In: Attorney-General NCfLRwtAot, editor. Nairobi; 2010.
11. . Maina BW, Mutua MM, Sidze EM. Factors associated with repeat induced abortion in Kenya. *BMC Public Health* 2015; **15**: 1048.
12. Ochako R, Izugbara C, Okal J, Askew I, Temmerman M. Contraceptive method choice among women in slum and non-slum communities in Nairobi, Kenya. *BMC Womens Health* 2016; **16**: 35.
13. Postabortion Care Consortium. PAC Model. 2015. http://pac-consortium.org/resources/pac-model/ (accessed November 12 2016).
14. Gemzell-Danielsson K, Fiala C, Weeks A. Misoprostol: first-line therapy for incomplete miscarriage in the developing world. *BJOG* 2007; **114**(11): 1337–9.
15. Weeks A, Alia G, Blum J, et al. A randomized trial of misoprostol compared with manual vacuum aspiration for incomplete abortion. *Obstet Gynecol* 2005; **106**(3): 540–7.
16. Blandine T, Ouattara AZ, Coral A, Hassane C, Clotaire H, Dao B, Lankoande J, Diop A, Blum J. Sublingual [corrected] misoprostol as first-line care for incomplete abortion in Burkina Faso. J.*Int J Gynaecol Obstet.* 2012;**119**(2):166-9.
17. African Population and Health Research Center, Ministry of Health, Kenya, Ipas, and Guttmacher Institute. *Incidence and Complications of Unsafe Abortion in Kenya: Key Findings of a National Study* Nairobi. Nairobi, Kenya, 2012.
18. Huber D, Curtis C, Irani L, Pappa S, Arrington L. Postabortion Care: 20 Years of Strong Evidence on Emergency Treatment, Family Planning, and Other Programming Components. *Glob Health Sci Pract.* 2016; **29**(3):481-94.20.

For peer review only - http://bmjopen.bmj.com/site/about/guidelines.xhtml

19. Fulton BD, Scheffler RM, Sparkes SP, Auh EY, Vujicic M, Soucat A. Health workforce skill mix and task shifting in low income countries: a review of recent evidence. *Hum Resour Health* 2011; **9**: 1.

20. Osur J, Baird TL, Levandowski BA, Jackson E, Murokora D. Implementation of misoprostol for postabortion care in Kenya and Uganda: a qualitative evaluation. *Glob Health Action* 2013; **6**: 1–11.

21. Renner RM, Brahmi D, Kapp N. Who can provide effective and safe termination of pregnancy care? A systematic review*. *BJOG* 2013; **120**(1): 23–31.

22. Cleeve A, Oguttu M, Ganatra B, et al. Time to act-comprehensive abortion care in east Africa. *Lancet Glob Health* 2016; **4**(9): e601–2.

23. Warriner IK, Wang D, Huong NT, et al. Can midlevel health-care providers administer early medical abortion as safely and effectively as doctors? A randomised controlled equivalence trial in Nepal. *Lancet* 2011; **377**(9772): 1155–61.

24. Olavarrieta CD, Ganatra B, Sorhaindo A, Karver TS, Seuc A, Villalobos A , García SG, Pérez M, Bousieguez M, Sanhueza P. Nurse versus physician-provision of early medical abortion in Mexico: a randomized controlled non-inferiority trial. *Bulletin of the World Health Organization* 2015;93:249-258

25. Klingberg-Allvin M, Cleeve A, Atuhairwe S, et al. Comparison of treatment of incomplete abortion with misoprostol by physicians and midwives at district level in Uganda: a randomised controlled equivalence trial. *Lancet* 2015; **385**(9985): 2392–8.

26. World Health Organization. Optimizing health worker roles to improve access to key maternal and newborn health interventions through task shifting. [Accessed May 19, 2015];2012 http://apps.who.int/iris/bitstream/10665/77764/1/9789241504843_eng.pdf. [PubMed] (accessed Feb 03 2017).

27. Grimes DA, Benson J, Singh S, et al. Unsafe abortion: the preventable pandemic. *Lancet* 2006; **368**(9550): 1908–19.

28. Shah IH, Ahman E, Ortayli N. Access to safe abortion: progress and challenges since the 1994 International Conference on Population and Development (ICPD). *Contraception* 2014; **90**(6 Suppl): S39–48.

29. Izugbara C, Egesa C, Okelo R. 'High profile health facilities can add to your trouble': Women, stigma and un/safe abortion in Kenya. *Soc Sci & Med* 2015; **141:** 9-18.

30. Piaggio G, Elbourne DR, Pocock SJ, Evans SJ, Altman DG, Group C. Reporting of noninferiority and equivalence randomized trials: extension of the CONSORT 2010 statement. *JAMA* 2012; **308**(24): 2594–604.

31. Turner KL, ed. Medical abortion training guide, 2nd edn. Chapel Hill, NC: Ipas, 2010.29.

32. WHO. Medical eligibility criteria for contraceptive use. 2016. http://www.who.int/reproductivehealth/publications/family_planning/MEC-5/en/ (accessed Feb 02 2017).

33. Gebreselassie H, Gallo MF, Monyo A, Johnson BR. The magnitude of abortion complications in Kenya. *BJOG*. 2005; **112**(9):1336.

For peer review only

Table 1: Background characteristics of participating PAC providers.

Characteristics of providers	Midwife n = 19 (51.4%)	Physician group n =18 (48.6%)	Total n = 37 (100%)
**Age**			
Mean (SD)	47.3 (6.5)	35.9 (10.0)	41.7 (10.1)
Median	50 (6.0)	30 (14.5)	44 (20)
Range	28–55	26–60	26–60
**Sex**			
Female	18 (94.7%)	6 (33.3%)	24 (64.9%)
Male	1 (5.3%)	12 (66.7%)	13 (35.1%)
**Years of professional practice**			
Mean (SD)	22.4 (7.9)	8.8 (8.1)	15.8 (10.5)
Median (IQR)	26 (8.0)	4 (15)	17 (22)
Range	1– 30	1–28	1–30
**Clinical experience in PAC (years)***			
Mean (SD)	2.7 (1.6)	8.4 (6.9)	5.5 (5.5)
Median (IQR)	3 (3.0)	4 1(4.25)	3 (2.5)
Range	0–5	0–25	0–25

Data are n (%), mean (SD), or median (IQR). *Before study start.

Table 2: Sociodemographic characteristics and reproductive history of participants* by provider

Characteristics of participants	Midwife	Physician	Total
**Age (years)**			
n	422	416	838
Mean (SD)	25.2 (5.9)	24.8 (5.4)	25.0 (5.6)
Range	14–45	12–41	12–45
14–19	60 (14%)	56 (14%)	116 (14%)
20–24	171 (40%)	167 (40%)	338 (40%)
25–29	101 (24%)	113 (27%)	214 (26%)
30–34	53 (13%)	51 (12%)	104 (12%)
35–39	28 (7%)	26 (6%)	54 (7%)
40–47	9 (2%)	3 (1%)	12 (1%)
**Marital status**			
n	433	426	859
Married or cohabiting	289 (67%)	292 (68%)	581 (68%)
Single	128 (30%)	124 (29%)	252 (29%)
Divorced/separated	9 (2%)	7 (2%)	16 (2%)
Widowed	7 (2%)	3 (1%)	10 (1%)
**Religion**			
n	433	426	859
Christian	427 (99%)	417 (98%)	844 (98%)
Muslim	6 (1%)	9 (2%)	15 (2%)
**Education**			
n	433	425	858
None	6 (1%)	2 (1%)	8 (1%)
Primary 1–4	11 (2%)	9 (2%)	20 (2%)
Primary 1–5	108 (25%)	121 (28%)	229 (27%)
Secondary	206 (48%)	186 (44%)	392 (46%)
Tertiary	102 (24%)	107 (25%)	209 (24%)
**Occupation**			
n	431	423	854
Unemployed	210 (49%)	197 (47%)	407 (48%)
Formal employment	73 (17%)	75 (18%)	148 (17%)

For peer review only - http://bmjopen.bmj.com/site/about/guidelines.xhtml

Self-employed	148 (34%)	151 (36%)	299 (35%)
**Gestational age based on clinical examination (weeks)**			
n	433	426	859
Mean (SD)	9.4 (2.2)	9.7 (2.1)	9.6 (2.2)
Range	1–12	3–12	1–12
**Number of previous pregnancies**			
n	433	425	858
Mean (SD)	1.75 (1.8)	1.8 (1.6)	1.8 (1.7)
Range	0–12	0–8	0–12
0	135 (31%)	101 (24%)	236 (27%)
1	94 (22%)	110 (26%)	204 (24%)
2–3	136 (31%)	157 (37%)	293 (34%)
4–5	58 (13%)	50 (11%)	108 (13%)
6–8	7 (2%)	7 (2%)	14 (2%)
9–12	3 (1%)	0	3
**Parity (live birth)**			
n	431	425	856
Mean (SD)	1.1 (1.4)	1.1 (1.3)	1.1 (1.3)
Range	0-10	0-7	0-10
0	205 (48%)	178 (42%)	383 (45%)
1	105 (24%)	111 (26%)	216 (25%)
2-3	94 (22%)	115 (27%)	209 (24%)
4-5	23 (5%)	18 (4%)	41 (5%)
6-8	3 (1%)	3 (1%)	6 (1%)
9-11	1	0	1

Data are n (%) unless otherwise stated.
*Includes women lost to follow-up.
Internal drop-out range between 0–11 (0–2.6%), among the midwife and physician group, respectively.

Table 3: Outcome of treatment of incomplete abortion by provider

	Midwife	Physician	Risk difference (95% CI)	Adjusted difference (95% CI)*
Randomised and received intervention	446	444		
Per-protocol	409	401		
Complete abortion	390 (95.4%)	378 (94.3%)	1.0% (–4.1 to 2.0)	1.0% (–4.1 to 2.2)

*Adjusted for facility, age (<25 vs ≥25 years), marital status (single [single, widowed, divorced, separated] vs married or cohabiting), education (none or primary 1–8 vs secondary or tertiary), number of previous pregnancies (0 vs >1), and parity (para 0 vs ≥1).

Table 4: Secondary outcomes, symptoms following the treatment, by provider.

Secondary outcomes	Midwife	Physician	Total
**Bleeding since treatment**			
**N**	408	401	806
Less than normal menstrual bleeding	274 (67%)	268 (67%)	542 (67%)
Same as normal menstrual bleeding	78 (19%)	70 (18%)	148 (19%)
Havier than normal menstrual	56 (14%)	60 (15%)	116 (14%)

For peer review only - http://bmjopen.bmj.com/site/about/guidelines.xhtml

bleeding			
**Days bleeding**			
N	396	389	785
Mean	4.2 (2.0)	4.08 (1.7)	4.1 (2.0)
Range	1–14	1–10	1–14
**Pain following treatment**			
N	407	399	806
None	86 (21%)	84 (20%)	170 (21%)
Mild	200 (49%)	194 (49%)	394 (49%)
Moderate	81 (20%)	76 (19%)	157 (20%)
Severe	40 (10%)	45 (11%)	85 (10%)
**Unscheduled visit**			
N	403	397	800
Yes	20 (5%)	26 (6%)	46 (6%)
Vaginal bleeding or abdominal pain, or both	11 (3%)	18 (4%)	29 (4%)
Chill/fever or dizziness, or both	6 (1%)	6 (1%)	12 (1%)
**Acceptance of the method**			
How did you perceive the treatment			
N	407	398	801
As expected	121 (30%)	130 (32%)	251 (31%)
Worse than expected	29 (7%)	27 (7%)	56 (7%)
Easier than expected	257 (63%)	241 (61%)	498 (62%)
Felt safe			
N	400	399	799
Yes	383 (96%)	378 (95%)	779 (97%)
No	17 (4%)	21 (4%)	38 (3%)
Will recommend the treatment to a friend			
N	400	399	799
Yes	383 (96%)	378 (95%)	761 (95%)
No	17 (4%)	21 (5%)	38 (5%)

Data are n (%) unless otherwise stated.
Internal drop-out range between 0–11 (0–2.6%), among the midwife and physician group, respectively.

Table 5: Contraceptive uptake, by provider type.

**Contraceptive uptake**	**Midwife**	**Physician**	**Total**
**Received contraceptive counselling**			
N	405	401	806
Yes	395 (98%)	394 (98%)	789 (98%)
No	10 (2%)	7 (2%)	17 (2%)
**Accepted a contraceptive method**			
N	407	401	803
No	104 (26%)	91 (23%)	195 (24%)
Yes	303 (74%)	310 (77%)	613 (76%)
**What method did you chose**			
Injectable	118 (39%)	118 (38%)	236 (39%)
Pills	81 (27%)	85 (28%)	166 (27%)
Condoms	75 (25%)	76 (25%)	151 (25%)
Hormonal implants	24 (8%)	21 (7%)	45 (8%)
IUDs	3 (1%)	5 (2%)	8 (1%)
Permanent contraceptives	-	1	1

Data are n (%) unless otherwise stated.
Internal drop-out range between 0–11 (0–2.6%), among the midwife and physician group, respectively.

For peer review only - http://bmjopen.bmj.com/site/about/guidelines.xhtml

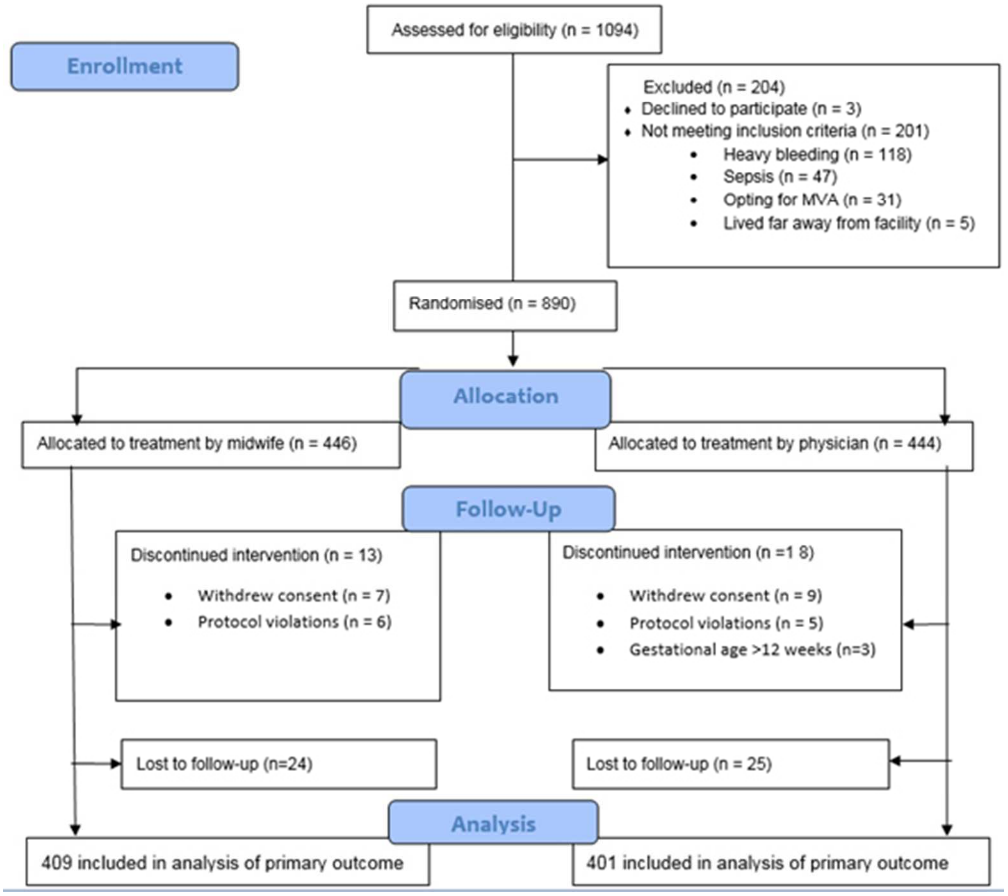

51x45mm (300 x 300 DPI)

only

Appendix 1. Side-effects following treatment of incomplete abortion, self-reported by participating women.

Self-reported side effects*	Midwife	Physician	Total
**N**	409	401	810
None	201 (49%)	206 (51%)	407 (50%)
Abdominal pain	166 (41%)	139 (35%)	305 (38%)
Chills	51 (12%)	46 (12%)	97 (12%)
Nausea	32 (8%)	32 (8%)	64 (8%
Diarrhoea	27 (7%)	22 (6%)	49 (6%)
Vomiting	12 (3%)	17 (4%)	29 (4%)
Foil smelling vaginal or cervical discharge	9 (2%)	10 (2%)	19 (2%)

Data are n (%). *Several options was allowed to obtain.

For peer review only

CONSORT 2010 checklist of information to include when reporting a randomised trial*

Section/Topic	Item No	Checklist item	Reported on page No
**Title and abstract**			
	1a	Identification as a randomised trial in the title	TIDieR
	1b	Structured summary of trial design, methods, results, and conclusions (for specific guidance see CONSORT for abstracts)	TIDieR
**Introduction**			
Background and objectives	2a	Scientific background and explanation of rationale	TIDieR
	2b	Specific objectives or hypotheses	192-95, p5
**Methods**			
Trial design	3a	Description of trial design (such as parallel, factorial) including allocation ratio	197-98, p8
	3b	Important changes to methods after trial commencement (such as eligibility criteria), with reasons	TIDieR
Participants	4a	Eligibility criteria for participants	237-44, p7
	4b	Settings and locations where the data were collected	TIDieR
Interventions	5	The interventions for each group with sufficient details to allow replication, including how and when they were actually administered	TIDieR
Outcomes	6a	Completely defined pre-specified primary and secondary outcome measures, including how and when they were assessed	300-22, p9
	6b	Any changes to trial outcomes after the trial commenced, with reasons	344, p10
Sample size	7a	How sample size was determined	325, p9
	7b	When applicable, explanation of any interim analyses and stopping guidelines	n/a
Randomisation:			
Sequence generation	8a	Method used to generate the random allocation sequence	248, p7
	8b	Type of randomisation; details of any restriction (such as blocking and block size)	248, p7
Allocation concealment mechanism	9	Mechanism used to implement the random allocation sequence (such as sequentially numbered containers), describing any steps taken to conceal the sequence until interventions were assigned	249-53, p7
Implementation	10	Who generated the random allocation sequence, who enrolled participants, and who assigned participants to interventions	251-52 p7, 271-77, p8

For peer review only - http://bmjopen.bmj.com/site/about/guidelines.xhtml

Blinding	11a	If done, who was blinded after assignment to interventions (for example, participants, care providers, those assessing outcomes) and how	258-59, p7
	11b	If relevant, description of the similarity of interventions	
Statistical methods	12a	Statistical methods used to compare groups for primary and secondary outcomes	329-33, p9
	12b	Methods for additional analyses, such as subgroup analyses and adjusted analyses	329-33, p9
**Results**			
Participant flow (a diagram is strongly recommended)	13a	For each group, the numbers of participants who were randomly assigned, received intended treatment, and were analysed for the primary outcome	Figure 1
	13b	For each group, losses and exclusions after randomisation, together with reasons	Figure 1
Recruitment	14a	Dates defining the periods of recruitment and follow-up	288, p8, 359, p10
	14b	Why the trial ended or was stopped	na
Baseline data	15	A table showing baseline demographic and clinical characteristics for each group	Table 1
Numbers analysed	16	For each group, number of participants (denominator) included in each analysis and whether the analysis was by original assigned groups	Fig1, Table 1-5
Outcomes and estimation	17a	For each primary and secondary outcome, results for each group, and the estimated effect size and its precision (such as 95% confidence interval)	Table 3
	17b	For binary outcomes, presentation of both absolute and relative effect sizes is recommended	
Ancillary analyses	18	Results of any other analyses performed, including subgroup analyses and adjusted analyses, distinguishing pre-specified from exploratory	Table 3+line 374-77, 391- 94, p11
Harms	19	All important harms or unintended effects in each group (for specific guidance see CONSORT for harms)	Appendix1
**Discussion**			
Limitations	20	Trial limitations, addressing sources of potential bias, imprecision, and, if relevant, multiplicity of analyses	410-21, p12
Generalisability	21	Generalisability (external validity, applicability) of the trial findings	408-09, p12
Interpretation	22	Interpretation consistent with results, balancing benefits and harms, and considering other relevant evidence	433-37, p13
**Other information**			
Registration	23	Registration number and name of trial registry	350, p10
Protocol	24	Where the full trial protocol can be accessed, if available	Attached
Funding	25	Sources of funding and other support (such as supply of drugs), role of funders	512-15, p15

For peer review only

For peer review only - http://bmjopen.bmj.com/site/about/guidelines.xhtml

*We strongly recommend reading this statement in conjunction with the CONSORT 2010 Explanation and Elaboration for important clarifications on all the items. If relevant, we also recommend reading CONSORT extensions for cluster randomised trials, non-inferiority and equivalence trials, non-pharmacological treatments, herbal interventions, and pragmatic trials. Additional extensions are forthcoming: for those and for up to date references relevant to this checklist, see www.consort-statement.org.

For peer review only

TIDieR
Template for Intervention Description and Replication

The TIDieR (Template for Intervention Description and Replication) Checklist*:

Information to include when describing an intervention and the location of the information

Item number	Item	Where located ** Primary paper (page or appendix number)	Other † (details)
	**BRIEF NAME**		
**1.**	Provide the name or a phrase that describes the intervention.	Page 1, Title	______________
	**WHY**		
**2.**	Describe any rationale, theory, or goal of the elements essential to the intervention.	Line 172-181, p 4-5	____________
	**WHAT**		
**3.**	Materials: Describe any physical or informational materials used in the intervention, including those provided to participants or used in intervention delivery or in training of intervention providers. Provide information on where the materials can be accessed (e.g. online appendix, URL).	Line 209-13, p6, line 261-270, p7	____________
**4.**	Procedures: Describe each of the procedures, activities, and/or processes used in the intervention, including any enabling or support activities.	Line 275-288, p8.	____________
	**WHO PROVIDED**		
**5.**	For each category of intervention provider (e.g. psychologist, nursing assistant), describe their expertise, background and any specific training given.	Line 265-70, p7 +Table 1	____________
	**HOW**		
**6.**	Describe the modes of delivery (e.g. face-to-face or by some other mechanism, such as internet or telephone) of the intervention and whether it was provided individually or in a group.	Line 279, p8	____________
	**WHERE**		

For peer review only - http://bmjopen.bmj.com/site/about/guidelines.xhtml

**7.**	Describe the type(s) of location(s) where the intervention occurred, including any necessary infrastructure or relevant features.	Line 199-209, p5-6, 261-65, p7, 275-78 p8	__________
	**WHEN and HOW MUCH**		
**8.**	Describe the number of times the intervention was delivered and over what period of time including the number of sessions, their schedule, and their duration, intensity or dose.	Line 283-288, p8	__________
	**TAILORING**		
**9.**	If the intervention was planned to be personalised, titrated or adapted, then describe what, why, when, and how.	N/A	__________
	**MODIFICATIONS**		
**10.‡**	If the intervention was modified during the course of the study, describe the changes (what, why, when, and how).	Line 216 p6, 343-44, p10	__________
	**HOW WELL**		
**11.**	Planned: If intervention adherence or fidelity was assessed, describe how and by whom, and if any strategies were used to maintain or improve fidelity, describe them.	Line 255-58, 292-99 p8	__________
**12.‡**	Actual: If intervention adherence or fidelity was assessed, describe the extent to which the intervention was delivered as planned.	Table 3 and 4	__________

** **Authors** - use N/A if an item is not applicable for the intervention being described. **Reviewers** – use '?' if information about the element is not reported/not sufficiently reported.

† If the information is not provided in the primary paper, give details of where this information is available. This may include locations such as a published protocol or other published papers (provide citation details) or a website (provide the URL).

‡ If completing the TIDieR checklist for a protocol, these items are not relevant to the protocol and cannot be described until the study is complete.

* We strongly recommend using this checklist in conjunction with the TIDieR guide (see *BMJ* 2014;348:g1687) which contains an explanation and elaboration for each item.

* The focus of TIDieR is on reporting details of the intervention elements (and where relevant, comparison elements) of a study. Other elements and methodological features of studies are covered by other reporting statements and checklists and have not been duplicated as part of the TIDieR checklist. When a **randomised trial** is being reported, the TIDieR checklist should be used in conjunction with the CONSORT statement (see www.consort-statement.org) as an extension of **Item 5 of the CONSORT 2010 Statement.** When a **clinical trial protocol** is being reported, the TIDieR checklist should be used in conjunction with the SPIRIT statement as an extension of **Item 11 of the SPIRIT 2013**

For peer review only - http://bmjopen.bmj.com/site/about/guidelines.xhtml

For peer review only

Statement (see www.spirit-statement.org). For alternate study designs, TIDieR can be used in conjunction with the appropriate checklist for that study design (see www.equator-network.org).

For peer review only

For peer review only - http://bmjopen.bmj.com/site/about/guidelines.xhtml
